# GLIMMER: an interim subgroup analysis from an ongoing prospective study evaluating hyperspectral imaging for MGMT promoter methylation in gliomas

**DOI:** 10.1007/s11060-025-05340-2

**Published:** 2025-11-17

**Authors:** Johannes Wach, Ferdinand Weber, Tim Wende, Martin Vychopen, Alonso Barrantes-Freer, Annekatrin Pfahl, Hannes Köhler, Erdem Güresir

**Affiliations:** 1 Department of Neurosurgery, University Hospital Leipzig, Leipzig, Saxony, Germany; 2 Comprehensive Cancer Center Central Germany, Partner Site Leipzig, Leipzig, Saxony, Germany; 3 Paul-Flechsig-Institute of Neuropathology, University Hospital Leipzig, Leipzig, Saxony, Germany; 4 Innovation Center Computer Assisted Surgery, Faculty of Medicine, Leipzig University, 04103 Leipzig, Saxony, Germany; 5 Department of Neurosurgery, University Hospital Leipzig, Leipzig University, Liebigstraße 20, 04103 Leipzig, Germany

**Keywords:** Glioblastoma, Glioma surgery, Hyperspectral imaging, MGMT promoter methylation, Intraoperative imaging, Supramaximal resection, Optical biomarkers

## Abstract

**Background:**

MGMT promoter methylation is of importance in glioma regarding prognosis and management. Non-methylated MGMT glioblastoma patients seem to benefit more from gross total resection. MGMT status is not ultra-rapidly available in the operating room. The presents study is the first aiming to evaluate whether the novel imaging technique intraoperative hyperspectral imaging (HSI) can predict MGMT promoter methylation status in glioma patients using a novel optical scoring system.

**Methods:**

This was a prospective subgroup analysis of 25 glioma patients enrolled in a single-center observational study. Patients underwent in-vivo HSI (spectral range: 500–1000 nm) targeting non-contrast-enhancing tumor regions during resection. Two optical parameters—tissue water index (TWI) and organ hemoglobin index (OHI)—were extracted and combined into a novel three-point GLIMMER score. Primary endpoint was MGMT promoter methylation status determined by pyrosequencing. Diagnostic performance of the GLIMMER score was measured by AUC, sensitivity, and specificity. A subgroup analysis focused on IDH-wild-type glioblastoma (*n* = 16).

**Results:**

OHI ≤ 0.606 and TWI ≥ 0.501 were significantly associated with MGMT promoter methylation. The combined GLIMMER score achieved an AUC of 0.95 (95% CI, 0.87–1.00) with 94.7% sensitivity and 83.3% specificity. In the glioblastoma subgroup, the AUC was 0.97, with 100% sensitivity and 75% specificity. Patients scoring ≥ 1 point had significantly higher MGMT methylation (31.5%) than those scoring 0 points (7.9%; *p* < 0.001).

**Conclusions:**

The GLIMMER score suggests potential ultra-rapid, non-invasive intraoperative estimation of MGMT promoter methylation with high diagnostic performance. These findings necessitate further validation with in-vivo HSI-guided biopsies to guide future personalized resection strategies in glioma patients.

**Clinical trial number:**

German Clinical Trials Register (DRKS, Trial number: DRKS00036771, Registration date: 05.05.2025).

**Graphical Abstract:**

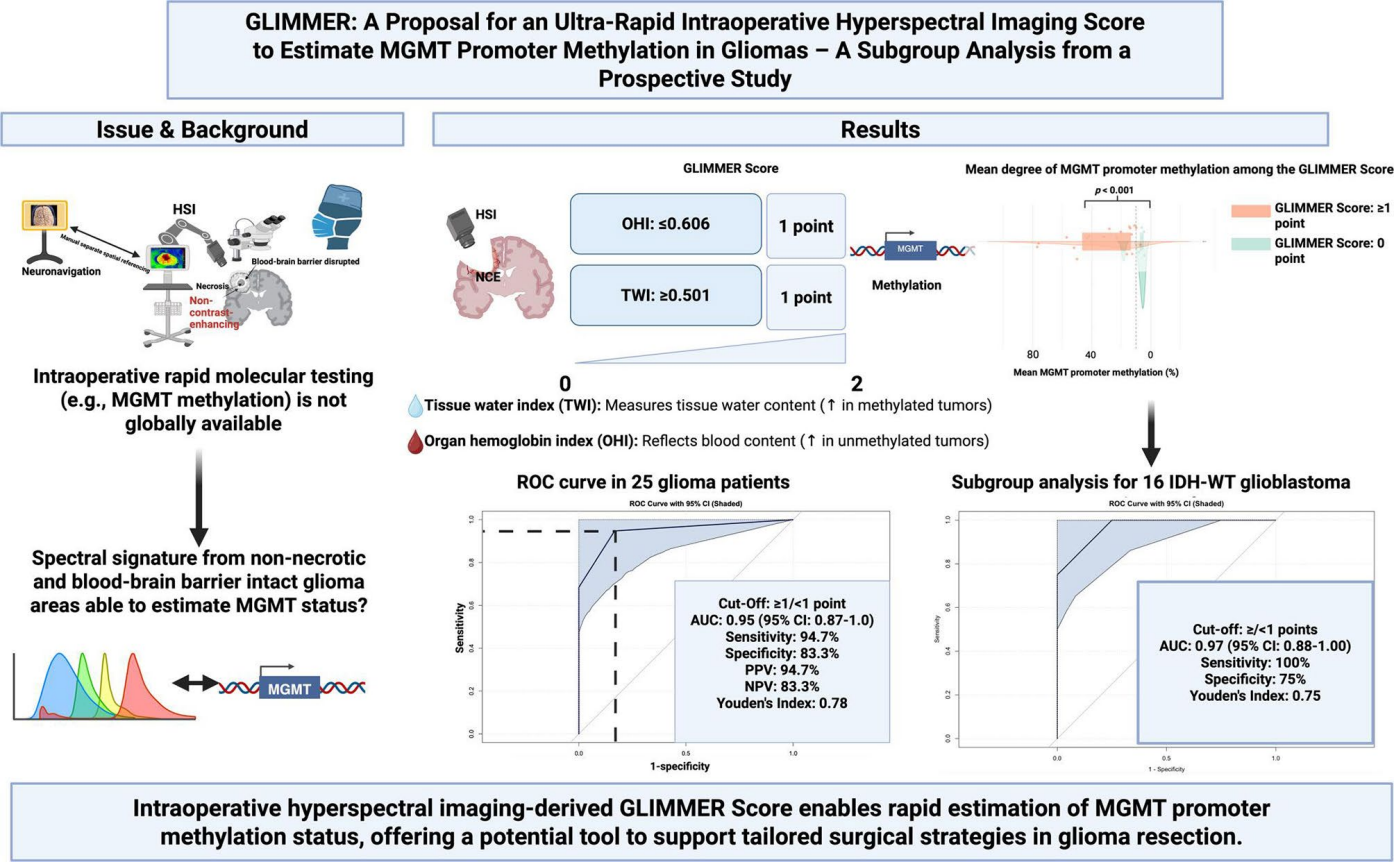

**Supplementary Information:**

The online version contains supplementary material available at 10.1007/s11060-025-05340-2.

## Introduction

Maximal safe resection remains the cornerstone of glioblastoma (GB) management, significantly impacting overall survival (OS) [1, 2]. This effect is particularly pronounced in patients with unmethylated O6-methylguanine-DNA methyltransferase (MGMT) promoters, where aggressive cytoreduction confers prognostic advantage [3]. Recent efforts underscore that the volume of residual tumor—especially non-contrast enhancing (NCE) regions—plays a critical role in outcomes in the era of supramarginal resection resection [4, 5]. The interaction between tumor biology and surgical strategy varies by DNA methylation subclass, as mesenchymal tumors benefit less from extensive resection than RTK I/II types [6].

Consequently, a rapid intraoperative assessment of MGMT methylation status could inform surgical strategy, balancing oncological benefit and neurological risk. However, currently available molecular platforms, such as nanopore-based sequencing workflows (e.g., Rapid-CNS2), while promising and emergingly under investigation, require at least 30–40 min and involve technical infrastructure [7].

*To address this limitation*,* our previous work demonstrated the feasibility of hyperspectral imaging (HSI) as a novel non-invasive*,* contrast agent-free method to assess glioma tissue properties intraoperatively. We identified correlations between HSI-derived indices*,* such as the tissue water index (TWI)*,* and FLAIR hyperintensity of NCE tumor portions*,* suggesting its potential in delineating these tumor zones* [8].

*In the current study*,* we focused specifically on solid*,* NCE tumor portions without necrosis or blood-brain barrier disruption*, *to reduce confounding bias from heterogeneous optical properties*,* which could interfere reflectance measurements* [9, 10]. *This methodological decision aligns with recent findings that the proportion of non-enhancing tumor is associated with MGMT promoter methylation* [11].

This subgroup analysis from an ongoing prospective study evaluates whether intraoperative in-vivo HSI enables rapid estimation of MGMT promoter methylation, potentially offering a practical, time-efficient tool for personalized decision-making in glioma surgery.

## Methods

### Study design

This subgroup analysis is part of the ongoing prospective proof-of-concept study “SPECTRE (**S**pectral **P**erfusion and **E**valuation of **C**erebral **T**issue via **R**eflectance-based **E**xamination study)” investigating in-vivo HSI in cranial neuro-oncological and neurovascular surgery. All trial documents, including the protocol, were approved by the Ethics Committee of the medical faculty of Leipzig University (047/24-ek) before recruitment. The study is registered at the German Clinical Trials Register (DRKS; trial number: DRKS00036771; registration date: 05.05.2025, www.drks.de). The full study protocol is provided in Supplementary Material 1. Reporting follows STROBE guidelines (Supplementary Material 2) [12].

### Inclusion criteria

Only hyperspectral images from solid, non-necrotic, and NCE glioma regions were used, analogous to established intraoperative HSI studies. This cohort for subgroup analysis was considered to reduce spectral heterogeneity due to interference from necrotic fluid [13]. All these patients were ≥ 18 years old and underwent neuronavigation- and 5-aminolevulinic acid (5-ALA) guided glioma resection with in-vivo HSI.

### HSI setup and protocol

The CE-certified TIVITA^®^ Tissue HSI system (Diaspective Vision GmbH, Salzhaff, Germany) enables non-invasive, contrast-free intraoperative imaging with qualitative and quantitative analysis of four parameters between 500 and 1000 nm. tissue oxygenation (StO₂), near-infrared perfusion index (NIR), organ hemoglobin index (OHI), and tissue water index (TWI). Each parameter is derived from specific wavelength bands detailed in Supplementary Table 1. StO₂ represents oxygen saturation in superficial tissue (reported as %), while NIR, OHI, and TWI are given as index values (0–100%) displayed as color-coded maps. The indices quantify oxygenation, perfusion, hemoglobin concentration, and water content through reflectance-based spectral analysis, allowing real-time assessment of tissue physiology without the need for contrast agents or additional instrumentation [14]. Imaging is performed from a fixed 50 cm distance using a mobile cart system with an integrated computer as previously reported [8, 15]. Each image is acquired within 10 s. Mean values from defined ROIs in NCE regions were analyzed. Intraoperative HSI followed a standardized sequence: post-dural opening, tumor surface, resection margins, and ex vivo specimens. Spatial referencing via neuronavigation markers (Brainlab Curve) enabled correlation with preoperative MRI (Supplementary Fig. 1). ROIs were sampled from distinct, neuronavigation-referenced, non-enhancing solid tumor areas and matched with focus of the HSI camera to reduce heterogeneity.

### Surgical workflow

Glioma patients received 5-ALA (20 mg/kg) orally 3 h preoperatively. Resection under white-light microscopy was neuronavigation-guided (BrainLAB Curve). In 22/25 NCE resections, intraoperative 3 T MRI (Siemens Magnetom Vida) assessed extent and corrected brain shift. As all received 5-ALA, HSI was performed under white light before blue-light excitation to avoid PpIX interference.

### Clinical and imaging data recording

Patients’ demographics, medication, and neurological outcomes were recorded. Non–contrast-enhancing (NCE) tumor volumes were semi-automatically segmented using SmartBrush (Brainlab AG, Munich, Germany). T2-FLAIR signal intensity ratios of NCE regions were calculated as previously described with ImageJ (National Institutes of Health) [8, 16, 17]. Corresponding ROIs were manually placed on apparent diffusion coefficient (ADC) maps to match intraoperative HSI acquisition sites. Only solid tumor tissue was analyzed; cystic, necrotic, and hemorrhagic areas were excluded. For diffusion quantification, a matching ROI was drawn in contralateral normal-appearing brain on the ADC map, and tumor-to-normal ADC ratios were calculated [18].

### Histopathology and molecular analyses

Neuropathological grading was performed according to the fifth edition of the classification of central nervous systems (CNS) tumors of the World Health Organization (WHO) [19]. MGMT data and quantification were obtained using pyrosequencing, and mean values were used for statistical analysis. Gliomas were categorized as either MGMT promoter methylated or unmethylated based on a cutoff of ≤ 8% across CpG sites 74–80, consistent with thresholds reported in previous comparative analyses between pyrosequencing and non-quantitative PCR [20]. Genome-wide DNA methylation profiling was performed using the Infinium MethylationEPIC v1.0 BeadChip (850k array, Illumina, San Diego, USA). Methylation profiles were compared with reference classes from the German Cancer Research Center (DKFZ) database (www.molecularneuropathology.org) to determine methylation class and derive copy number variation (CNV) profiles [21, 22]. CNV analysis enabled assessment of 1p/19q codeletion and chromosomal alterations. *IDH1/2* and *TERT* promoter mutations were identified by targeted DNA sequencing using the QIASeq Targeted DNA Panel for Solid Tumors (Qiagen, Hilden, Germany) and MiSeq sequencing (Illumina, San Diego, USA). Bioinformatic analysis and variant calling were conducted with Seamless NGS Software (EcSeq).

### Statistics

The primary endpoint was MGMT promoter methylation, dichotomized as methylated or unmethylated using a validated pyrosequencing threshold (≤ 8% mean methylation across CpG sites 74–80). Continuous and categorical variables were compared using *t*-tests or Fisher’s exact tests (two-sided). Data visualization included raincloud, box, scatter, and bubble plots illustrating MGMT methylation across HSI-derived strata and molecular subgroups. Receiver operating characteristic (ROC) curves assessed the diagnostic performance of individual and combined HSI parameters. Area under the curve (AUC), sensitivity, specificity, positive predictive value (PPV), and negative predictive value (NPV) were calculated. The two most discriminative HSI parameters were integrated into a three-point composite score, with thresholds derived from optimal ROC cut-offs. Diagnostic accuracy was defined as the proportion of correctly classified cases using MGMT methylation as the reference. All tests were two-sided, and *p*-values < 0.05 were considered statistically significant. Statistical analyses were conducted using R (version 4.3.1). The GLIMMER score was constructed from the binary thresholds of OHI and TWI identified in ROC analyses. A positive result in either OHI or TWI was considered indicative of MGMT promoter methylation. The term “GLIMMER score” is used as a concise label for this two-parameter classification scheme. To further assess the association between continuous HSI parameters and MGMT promoter methylation, a logistic regression model including OHI and TWI as independent variables was computed. Model performance was evaluated using the area under the receiver operating characteristic curve (AUC) with 95% confidence intervals obtained by the DeLong method [23].

## Results

### Patient characteristics

Selection process from the total prospective database is presented in supplementary Fig. 2. Twenty-five patients were included (median age 53.0 years, IQR 41.0–63.5; 52.0% female). Median FLAIR volume of NCE tumor was 80.35 cm³ (IQR 32.3–129.0). Median in-vivo HSI values for NIR, StO₂, OHI, and TWI were 0.45, 0.51, 0.57, and 0.56, respectively. Most tumors were WHO grade 4 (68.0%). IDH wild-type occurred in 64.0%, MGMT promoter methylation in 76.0%, TERT mutation in 60.0%, and 1p/19q codeletion in 12.0% of cases. Further details are summarized in supplementary Tables 2 & supplementary Fig. 3. Demographic and MR-imaging characteristics were comparable between methylated and non-methylated groups, without significant differences across these parameters (see supplementary Table 3).

### Diagnostic accuracy of intraoperative in-vivo HSI parameters for MGMT promoter methylation

**Fig. 1:**
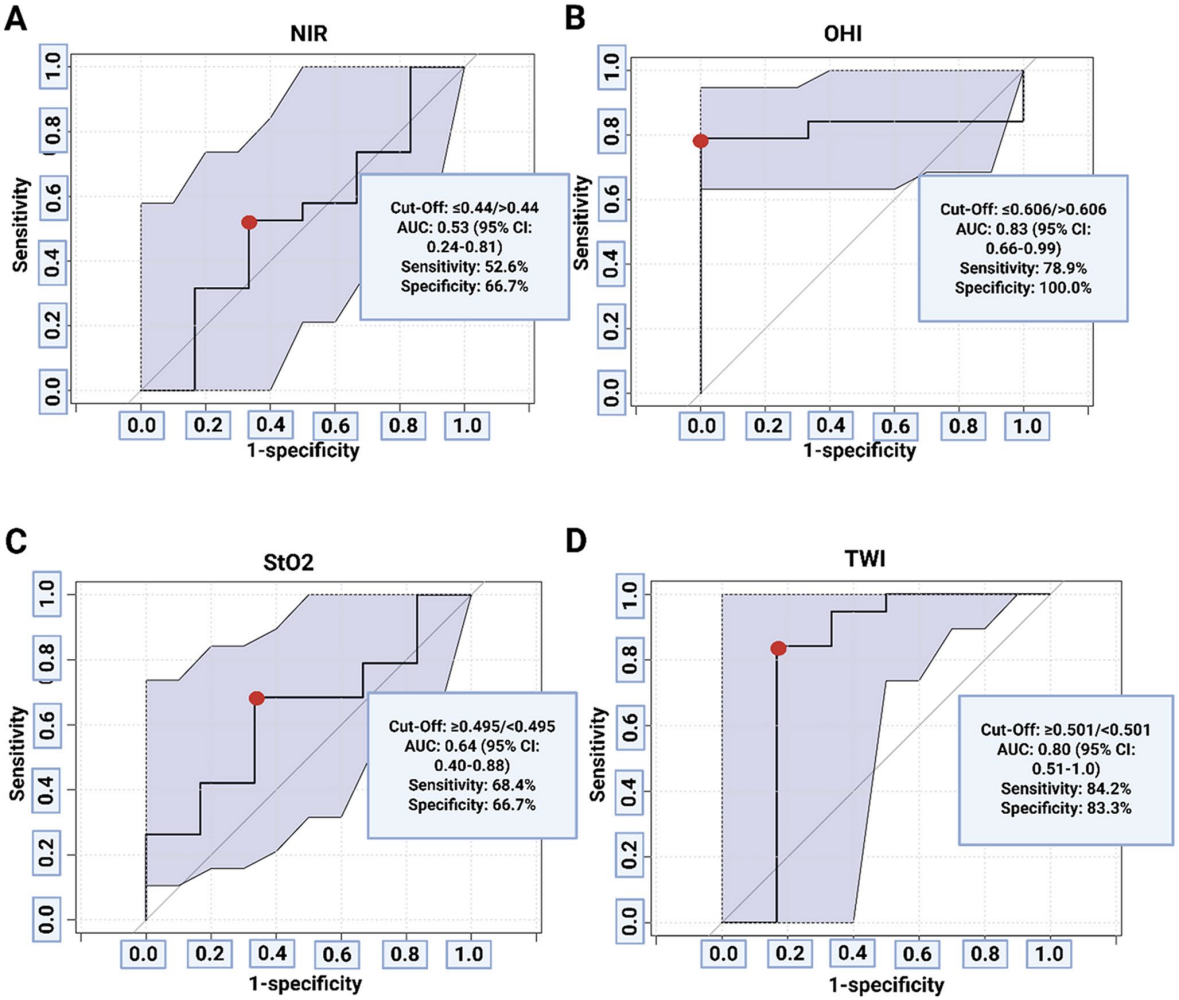
ROC curve analyses of individual hyperspectral imaging (HSI) parameters for the prediction of MGMT promoter methylation status. **(A)** Near-infrared (NIR) perfusion index, **(B)** Organ Hemoglobin Index (OHI), **(C)** Tissue Oxygen Saturation (StO₂), and **(D)** Tissue Water Index (TWI) were evaluated. Each curve includes 95% confidence intervals (shaded area) and the optimal classification threshold (red dot)

ROC curve analyses were performed to assess the diagnostic performance of intraoperative HSI indices in predicting MGMT promoter methylation (Fig. 1). Both OHI and TWI showed significant classification accuracy. OHI yielded an AUC of 0.83 (95%CI: 0.66–0.99), with 78.9% sensitivity and 100.0% specificity at a cut-off of ≤ 0.606. TWI achieved an AUC of 0.80 (95%CI: 0.51–1.0), with 84.2% sensitivity and 83.3% specificity at a threshold of ≥ 0.501.

**Fig. 2:**
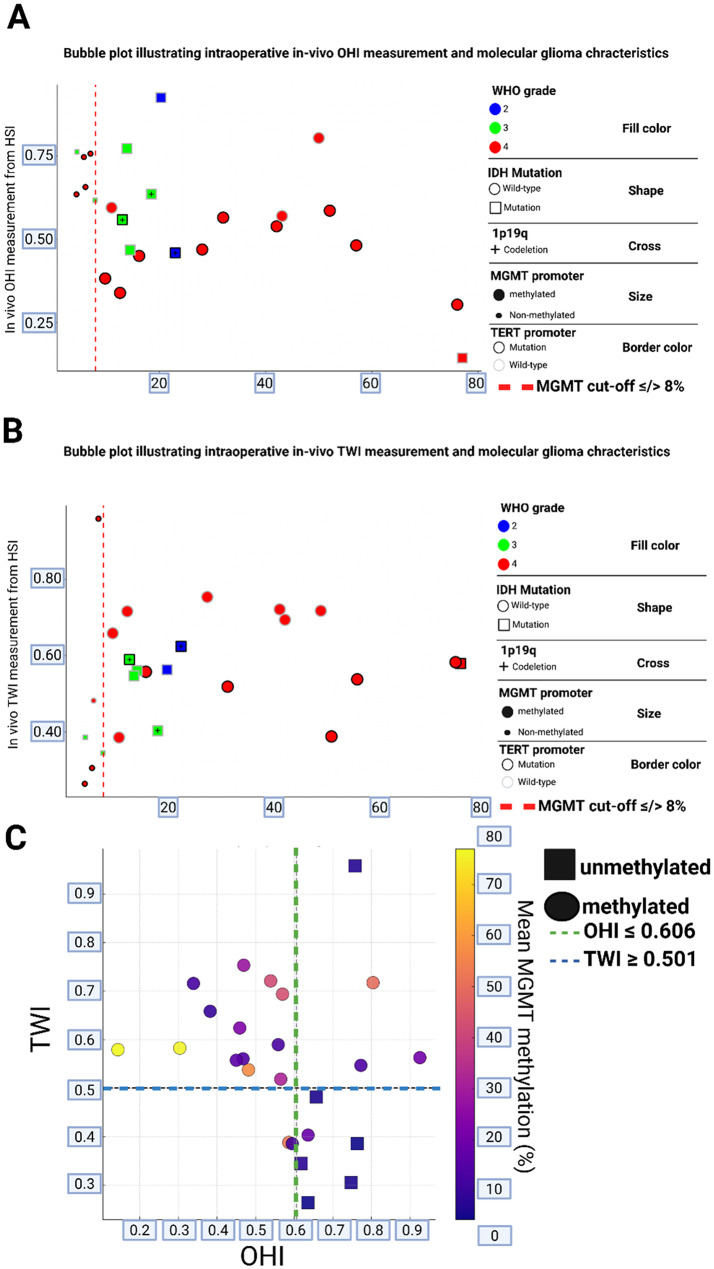
Bubble plots illustrating intraoperative in vivo HSI measurements in relation to MGMT promoter methylation and molecular glioma characteristics. **(A)** Organ Hemoglobin Index (OHI) and **(B)** Tissue Water Index (TWI) are plotted against the mean MGMT promoter methylation percentage. Each data point represents one patient and is encoded by WHO grade (fill color), IDH mutation status (shape), 1p19q codeletion (cross), MGMT promoter status (bubble size), and TERT promoter status (border color). The red dashed line denotes the methylation cut-off of ≤8%, as defined by Quillien et al. [20]. **(C)** 2D scatter plot displaying the relationship between OHI, TWI, and quantitative MGMT methylation (%). Color scale with heatmap indicates methylation level

To visualize the relationship between these significant HSI parameters and molecular characteristics, bubble plots were constructed (Fig. 2), incorporating WHO grade, IDH and TERT mutation status, 1p19q codeletion, and MGMT methylation percentage. Both OHI (Fig. 2A) and TWI (Fig. 2B) showed stratification trends consistent with methylation status. High OHI and low TWI values in non-methylated cases clustered in the scatter plot’s lower right quadrant (Fig. 2C).

To assess whether HSI parameters also reflect additional molecular alterations, exploratory ROC analyses were performed for IDH-1 and TERT promoter mutations (Supplementary Figs. 4 & 5). For IDH-1, AUC values ranged from 0.58 to 0.69, with StO₂ showing the highest discrimination (AUC = 0.69, 95%CI 0.47–0.90). For TERT, AUC values ranged from 0.60 to 0.76, with NIR demonstrating the highest AUC (0.76, 95%CI 0.55–0.96).

### Development and performance of the GLIMMER score

**Fig. 3:**
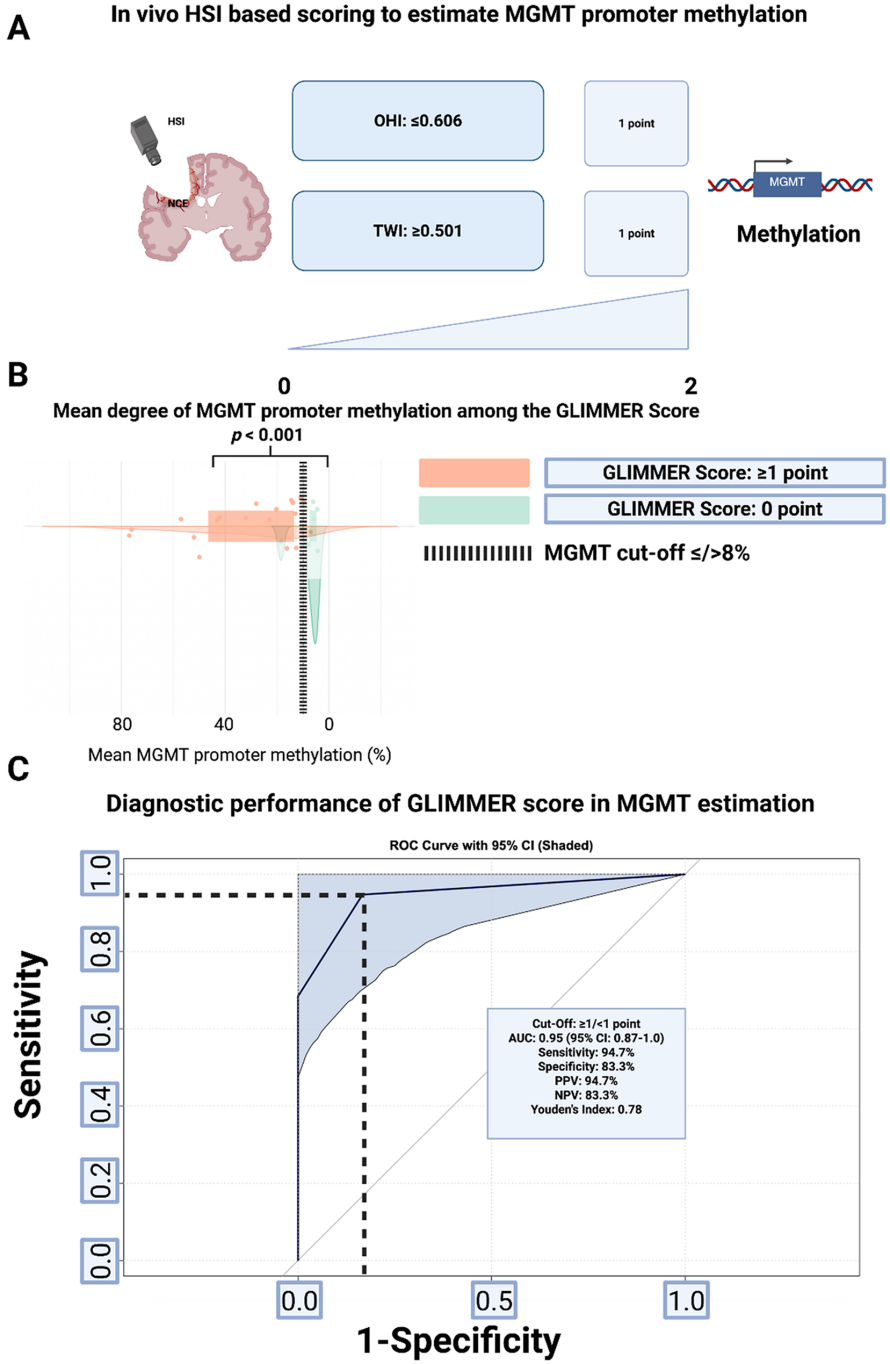
**(A)** Visual representation of the proposed *GLIMMER* Score based on the intraoperative in-vivo HSI parameters OHI and TWI estimating MGMT promoter methylation. **(B)** Raincloud plot comparing MGMT promoter methylation levels between score groups (0 vs. ≥1). The black dashed line labels the cut-off (≤/>8%) for MGMT methylation. **(C)** ROC curve evaluating the overall predictive accuracy of the proposed GLIMMER Score

To streamline intraoperative prediction of MGMT promoter methylation, a binary scoring system—termed the GLIMMER (**GLI**oma **M**GMT **M**ethylation **E**stimation via Ultra-Rapid **R**eflectance) Score—was developed using thresholds from ROC analysis. Combining OHI and TWI into a single score mitigates underdetection due to OHI’s lower sensitivity and overdetection from TWI’s slightly reduced specificity, enhancing diagnostic balance. One point each was assigned for OHI ≤ 0.606 and TWI ≥ 0.501 (Fig. 3A). Patients scoring ≥ 1 point were more likely to exhibit MGMT promoter methylation, with significantly higher mean methylation than those scoring 0 points (*p* < 0.001; Fig. 3B). Tumors scoring ≥ 1 point had a mean MGMT methylation of 31.5 ± 22.3%, versus 7.9 ± 5.4% in those scoring 0 points (*p* < 0.001). The combined score demonstrated high diagnostic accuracy for MGMT methylation status, with an AUC of 0.95 (95%CI: 0.87–1.0), sensitivity of 94.7%, and specificity of 83.3% (Fig. 3C).

**Fig. 4:**
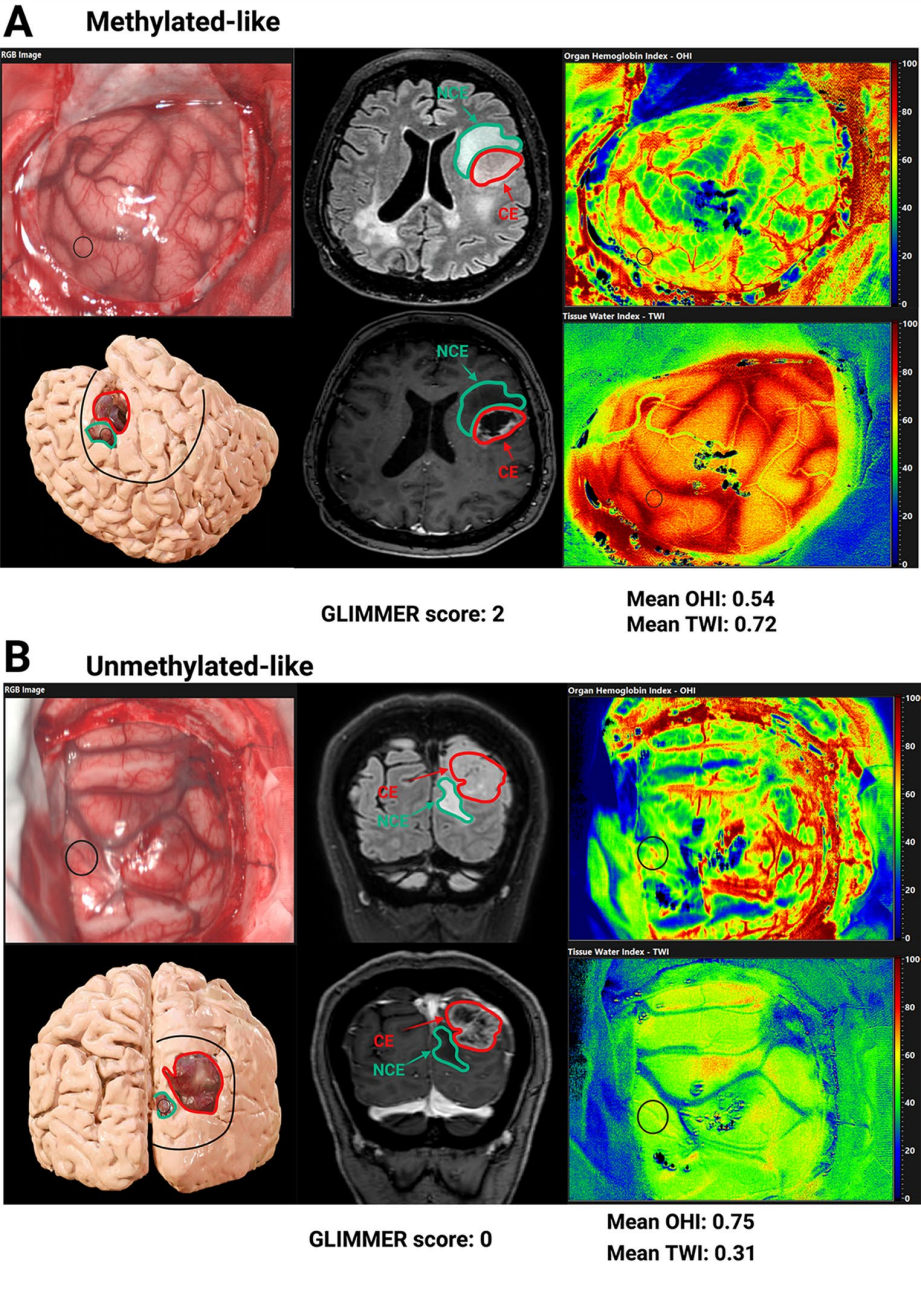
Illustrative examples of intraoperative hyperspectral imaging in glioblastoma with differing MGMT promoter methylation patterns. **(A)** Example of a “methylated-like” pattern: low OHI (mean: 0.54), high TWI (mean: 0.72), GLIMMER score of 2. **(B)** Example of an “unmethylated-like” pattern: high OHI (mean: 0.75), low TWI (mean: 0.31), GLIMMER score of 0. Each panel includes a T1 Gadolinium-enhanced MRI, FLAIR MRI with CE and NCE regions, a 3D anatomical model with durotomy marked, and RGB, OHI, and TWI images with ROIs labeled. The OHI and TWI maps are color-coded on a semi-quantitative scale, where blue indicates low values and red indicates high values

Positive and negative predictive values were 94.7% and 83.3%, respectively, with a Youden’s index of 0.78. The intraoperative in-vivo HSI score achieved 92% diagnostic accuracy, correctly classifying 23 out of 25 patients. An illustrative comparison between an unmethylated-like and a methylated-like subject is presented in Fig. 4. In the continuous-variable model, lower OHI (β = −7.65 ± 4.43, *p* = 0.084) and higher TWI (β = +4.90 ± 3.61, *p* = 0.176) were associated with MGMT promoter methylation. The model demonstrated good discrimination with an AUC of 0.86 (95%CI 0.69–1.00; supplementary Fig. 6).

### Subgroup analysis of the accuracy of the score in IDH-wild-type glioblastoma patients

**Fig. 5:**
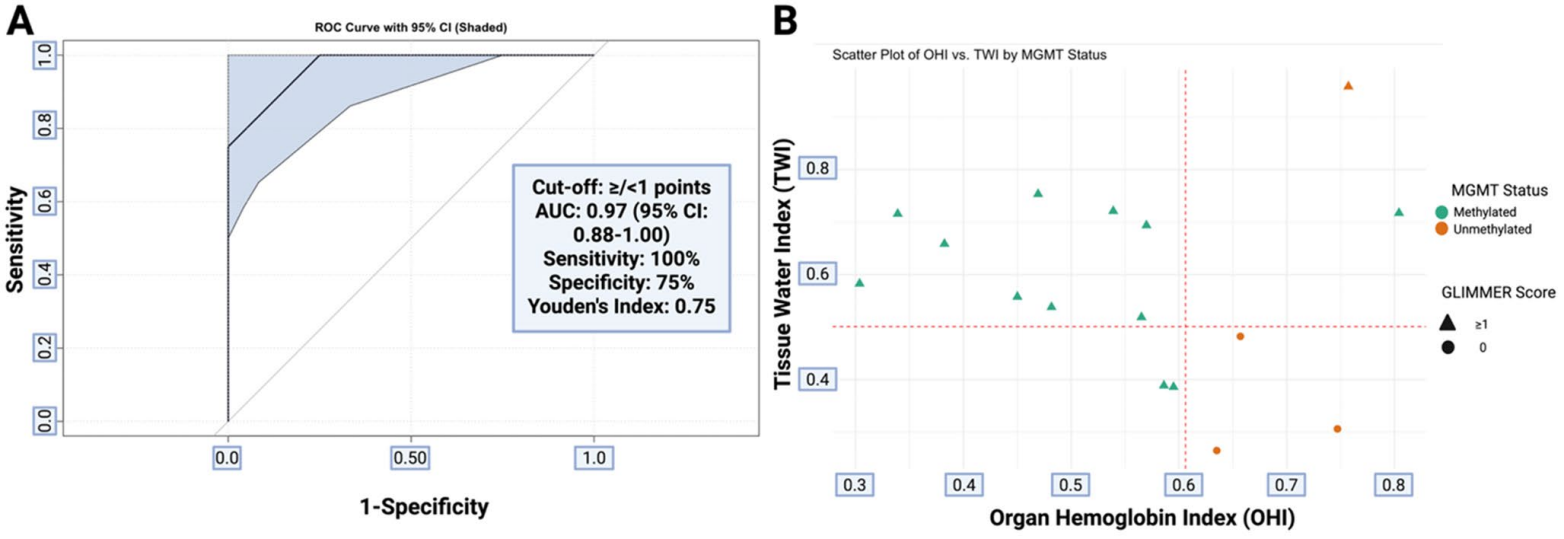
Subgroup analysis of IDH-wild-type glioblastoma patients (*n* = 16). **(A)** ROC curve of the GLIMMER score for MGMT promoter methylation. **(B)** 2D scatter plot of OHI vs. TWI. Points are color-coded by MGMT status and shaped by GLIMMER score. Red dashed lines indicate classification thresholds

To further validate the diagnostic performance of intraoperative HSI in high-grade tumors, we conducted a subgroup analysis of glioblastoma patients (*n* = 16). The GLIMMER score demonstrated promising diagnostic accuracy for MGMT methylation, with an AUC of 0.97 (95%CI: 0.88–1.00), 100% sensitivity, and 75% specificity (Fig. 5A). It correctly identified all methylated tumors (12/12, 100%) and misclassified only one of four unmethylated cases, resulting in 100% sensitivity and 75% specificity.

The Youden Index was 0.75. In the OHI vs. TWI scatter plot (Fig. 5B), methylated glioblastomas clustered below the OHI or above the TWI cut-offs, consistent with correct GLIMMER classification. This supports the robustness of the combined optical biomarker approach. In the glioblastoma subgroup, the GLIMMER score achieved 93.8% accuracy and correctly classified 15 of 16 patients.

## Discussion

To our knowledge, this is the first prospective in-vivo study directly linking HSI parameters to MGMT promoter methylation in glioblastoma. This investigation demonstrates the feasibility and diagnostic value of intraoperative in-vivo HSI for predicting MGMT status in GB patients. By focusing on solid, non-contrast-enhancing tumor components, we minimized confounding from necrosis and blood-brain barrier disruption. Our findings show that combining TWI and OHI into the GLIMMER score enables ultra-rapid, non-invasive molecular classification, with high diagnostic performance (AUC: 0.95 in all gliomas; 0.97 in GB) and 93.8% accuracy in the GB subgroup. This has significant implications for intraoperative decision-making and personalized GB surgery. Prior work by Black et al. [24] showed accurate IDH classification using ex-vivo HSI and machine learning but lacked real-time, in-vivo MGMT analysis.

### Extent of resection and MGMT methylation: surgical relevance

Maximal safe resection without residual contrast-enhancement is crucial in GB management, though its benefit varies by molecular subtype. Roder et al. [3] showed this impact is strongest in patients with unmethylated MGMT promoters, where even minimal residual tumor worsens outcomes. Similarly, Parker et al. [25] investigated 27,858 IDH-WT GB patients and found that MGMT methylation modulates the survival benefit of gross total resection even more in MGMT-non-methylated tumors or those not receiving temozolomide (e.g. elderly or frail patients). Novel stratification systems incorporating supramarginal resections categorize non-methylated GB patients into adverse prognostic groups, and intraoperative MGMT status might inform these approaches [26].

Ultra-rapid MGMT estimation via HSI may guide resection extent: in unmethylated cases, aggressive supratotal resection targeting NCE tumor could be prioritized if feasible, while in methylated tumors, neurological preservation may take precedence, aligning with differential treatment strategies and outcomes [3]. While maximal safe resection remains the goal in glioma surgery, intraoperative MGMT estimation may refine judgment in eloquent areas. Rapid identification of an unmethylated-like profile could justify cautious extension into non-enhancing tissue, whereas methylated tumors—more responsive to adjuvant therapy—may not warrant added surgical risk. The recent age-stratified analysis of the RANO resection group showed that survival benefit from supramaximal resection with low residual NCE tumor volume was mainly observed in MGMT-unmethylated glioblastoma [27]. Intraoperative MGMT estimation could help distinguish methylated tumors suitable for safe gross-total resection from unmethylated cases potentially benefiting from more extensive resection, within functional safety limits.

### Limitations of current intraoperative molecular techniques

Currently, no point-of-care platform enables MGMT status determination during early surgical phases. Frozen sections are unsuitable for epigenetic analysis, and standard methods like pyrosequencing or methylation-specific PCR require hours to days. Nanopore-based approaches (e.g., Rapid-CNS2) show promise with sub-hour classification but still demand complex sample preparation, technical expertise, and high-performance computing—requirements that exceed the capabilities of most operative environments, limiting their immediate applicability for intraoperative decision-making [7, 28].

In the recent ROBIN study, nanopore-derived MGMT status showed strong concordance with array-based results: 95.7% (44/46) prospectively and 86.2% (25/29) retrospectively, confirming its diagnostic potential despite the need for tissue and sequencing infrastructure [29]. In contrast, HSI offers a non-invasive, contrast-free approach detecting oxygenation, hemoglobin, and water content, potentially reflecting MGMT promoter methylation–related tissue differences.

### Diagnostic performance and biological plausibility of HSI markers

The diagnostic utility of HSI-derived parameters—TWI and OHI—for MGMT promoter methylation is supported by both performance and biological rationale. TWI reflects water content and indirectly cellular density. MGMT-methylated glioblastomas show higher ADC values on diffusion MRI, indicating lower cellularity [30]. In our cohort, methylated tumors showed elevated TWI values, consistent with diffusion profiles and increased water content.

OHI reflects hemoglobin absorption and local perfusion. MRI studies have shown that MGMT-methylated glioblastomas exhibit reduced perfusion and blood flow compared to unmethylated tumors [31, 32]. In the dynamic susceptibility-perfusion MRI study by Chida et al. [31], methylated glioblastomas had lower relative cerebral blood volume, attributed to decreased angiogenic activity. Similarly, Lu et al. [32] reported lower cerebral blood volume, simplified perfusion fraction, and microcirculation perfusion coefficient in methylated glioblastomas. The observed reduction of OHI in methylated tumors aligns with these perfusion findings, suggesting that HSI may approximate intraoperative vascular characteristics.

Together, TWI and OHI from HSI might provide a physiological snapshot of tumor microstructure and vascularization. Their combination in the GLIMMER score may enhance predictive accuracy by balancing sensitivity (TWI) and specificity (OHI), yielding an AUC of 0.95 overall and 0.97 in glioblastoma-only analysis. Thus, these spectral markers offer biologically meaningful, ultra-rapid intraoperative insight into MGMT status.

Beyond MGMT, exploratory analyses for IDH-1 and TERT mutations were conducted to evaluate whether HSI captures broader molecular information. The moderate performance for IDH-1 and the weaker, variable trends for TERT support that intraoperative HSI predominantly reflects metabolic and perfusion-related contrasts rather than purely genetic alterations. This observation aligns with prior evidence linking IDH mutation to altered tumor oxygenation and vascular architecture.

### Clinical utility and implications beyond surgery

Beyond surgical planning, MGMT status informs adjuvant therapy decisions. It is a well-established predictor of response to alkylating agents like temozolomide, and its role in radiosensitivity is increasingly recognized [33]. Preclinical and clinical studies show that MGMT promoter methylation enhances radiosensitivity, likely via impaired DNA repair [34]. Thus, accurate intraoperative MGMT assessment could guide novel strategies like intraoperative radiotherapy, especially in centers using protocols such as INTRAGO, where radiation follows immediately intra-cavitary after resection [35].

### Limitations

Despite promising results, intraoperative HSI remains limited by the lack of microscope integration, requiring manual navigation-based referencing and causing spatial inaccuracies. Future microscope integration could enable real-time, spatially registered imaging with higher precision also in deeper subcortical areas. As only non-enhancing regions were analyzed, applicability to enhancing tumor areas remains restricted. Edema, necrosis, and hemorrhage effects warrant larger studies. Misclassified cases likely reflect sampling error and intratumoral MGMT heterogeneity influencing optical–molecular correlations [36]. The high proportion of MGMT-methylated tumors (75%) in the entire cohort may reflect selection bias toward surgically accessible cases, despite the subgroup analysis of 16 IDH-wild-type glioblastomas.

### Outlook and future directions

This study presents the first in-vivo prospective evidence that HSI might be able to predict MGMT promoter methylation status in glioblastoma during surgery with high accuracy. The GLIMMER score provides a practical, quick, and time-efficient tool for intraoperative molecular guidance. Future steps include HSI-guided tissue sampling in a multicenter cohort with direct HSI-neuronavigation integration and external validation of these findings.

## Conclusions

In conclusion, our findings support the feasibility and diagnostic strength of a novel intraoperative in-vivo HSI-based scoring system as an ultra-rapid, non-invasive tool to estimate MGMT status. Integrating MGMT-specific optical biomarkers may enhance personalized neurosurgical strategies, potentially influencing immediate surgical decisions and long-term oncologic outcomes.

## Supplementary Information

Below is the link to the electronic supplementary material.

Supplementary Material 1

Supplementary Material 2

Supplementary Material 3

Supplementary Material 4

Supplementary Material 5

Supplementary Material 6

Supplementary Material 7

Supplementary Material 8

Supplementary Material 9

Supplementary Material 10

Supplementary Material 11

## Data Availability

The data sets generated and analyzed in the current study are available upon request from the corresponding author.
