# Supplementary material for: GLIMMER: an interim subgroup analysis from an ongoing prospective study evaluating hyperspectral imaging for MGMT promoter methylation in gliomas
Source: J Neurooncol. 2025 Nov 17;176(1):86. doi: 10.1007/s11060-025-05340-2 (PMC12628469; doi:10.1007/s11060-025-05340-2)

Supplementary figure 1: Intraoperative setup for hyperspectral imaging (HSI) using the TIVITA® Tissue system. HSI was performed at a fixed distance during defined surgical phases, focusing on solid, non-contrast-enhancing tumor regions. Neuronavigation was used for spatial referencing to preoperative MR imaging.

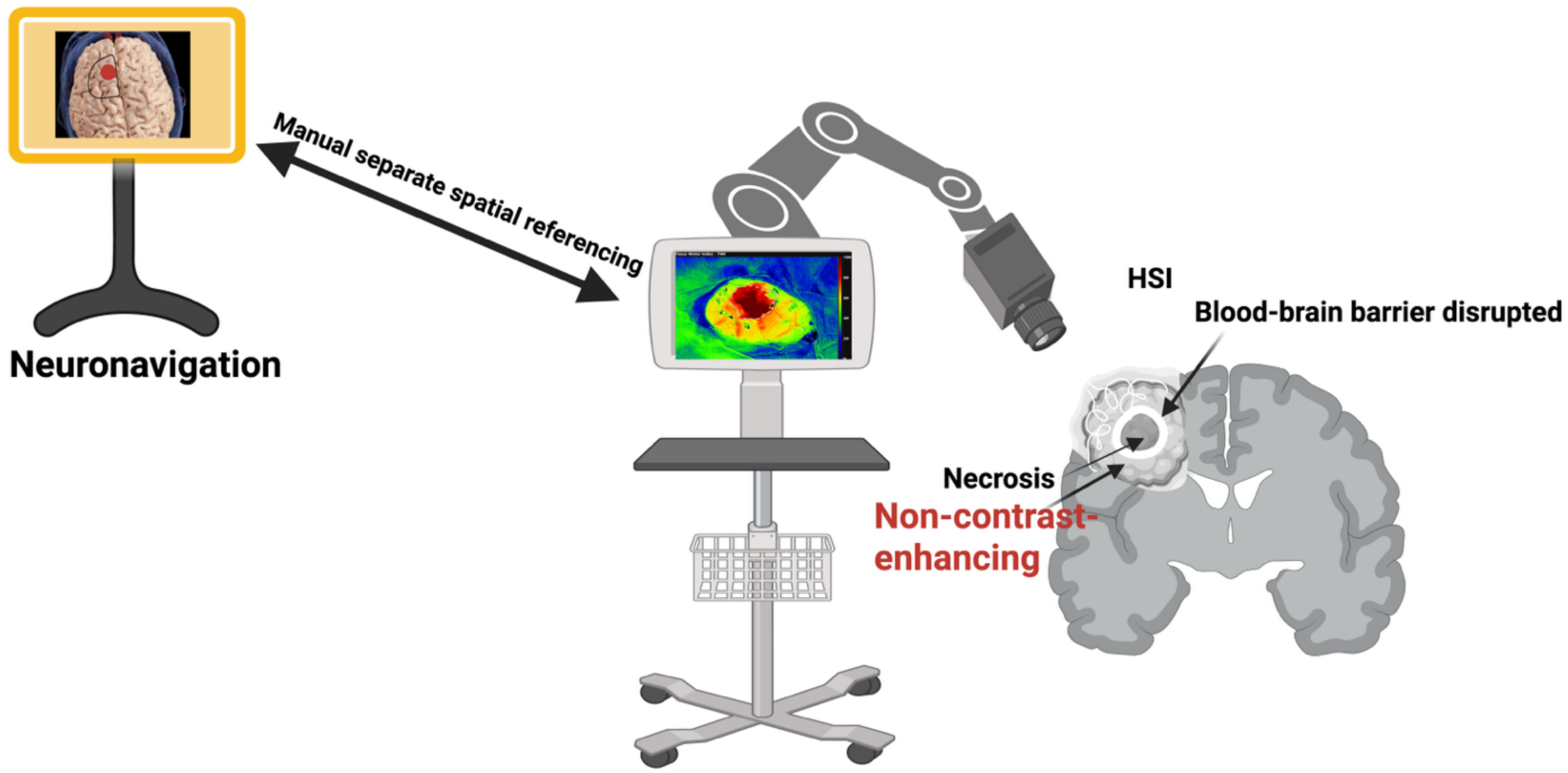

Supplement: Supplementary file 1 — Supplementary Material 1 [file 11060_2025_5340_MOESM1_ESM.pdf]
