# Supplementary material for: GLIMMER: an interim subgroup analysis from an ongoing prospective study evaluating hyperspectral imaging for MGMT promoter methylation in gliomas
Source: J Neurooncol. 2025 Nov 17;176(1):86. doi: 10.1007/s11060-025-05340-2 (PMC12628469; doi:10.1007/s11060-025-05340-2)

Supplementary figure 2. Between March 2024 and May 2025, intraoperative hyperspectral imaging (HSI) was performed in 141 patients undergoing surgery for neurooncological and neurovascular pathologies. Among these, 47 patients had gliomas who underwent surgical therapy via craniotomy. Of these, 25 patients had clearly traceable, non-contrast-enhancing regions (navigated including FLAIR sequences) that were both visualized intraoperatively with HSI and analyzed regarding MGMT promoter methylation. Twenty-two patients were excluded due to insufficient visualization with HSI or unavailability of matching with non-enhancing FLAIR areas during surgery.

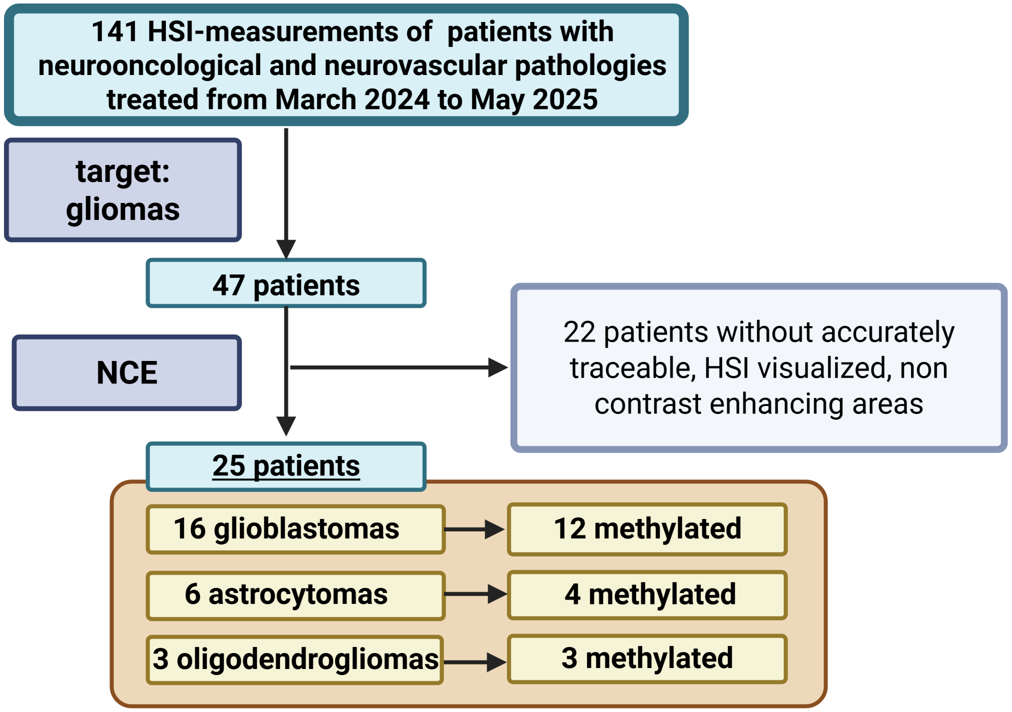

Supplement: Supplementary file 2 — Supplementary Material 2 [file 11060_2025_5340_MOESM2_ESM.pdf]
