# Supplementary material for: GLIMMER: an interim subgroup analysis from an ongoing prospective study evaluating hyperspectral imaging for MGMT promoter methylation in gliomas
Source: J Neurooncol. 2025 Nov 17;176(1):86. doi: 10.1007/s11060-025-05340-2 (PMC12628469; doi:10.1007/s11060-025-05340-2)

Supplementary Figure 3. Oncoprint of molecular and clinical features in 25 glioma patients. Each column represents an individual patient. Rows display: WHO grade (light gray = grade 2, gray = grade 3, black = grade 4), binary molecular alterations (blue = IDH mutation; green = MGMT promoter methylation; orange = TERT mutation; purple = 1p19q codeletion; red = CDKN2A/B homozygous deletion), and mean MGMT promoter methylation percentage (continuous grayscale gradient, white to black). White in binary rows indicates absence of the alteration. The color bar denotes the scale of mean MGMT methylation (%). X-axis indicates individual patients; Y-axis lists clinical and molecular variables.

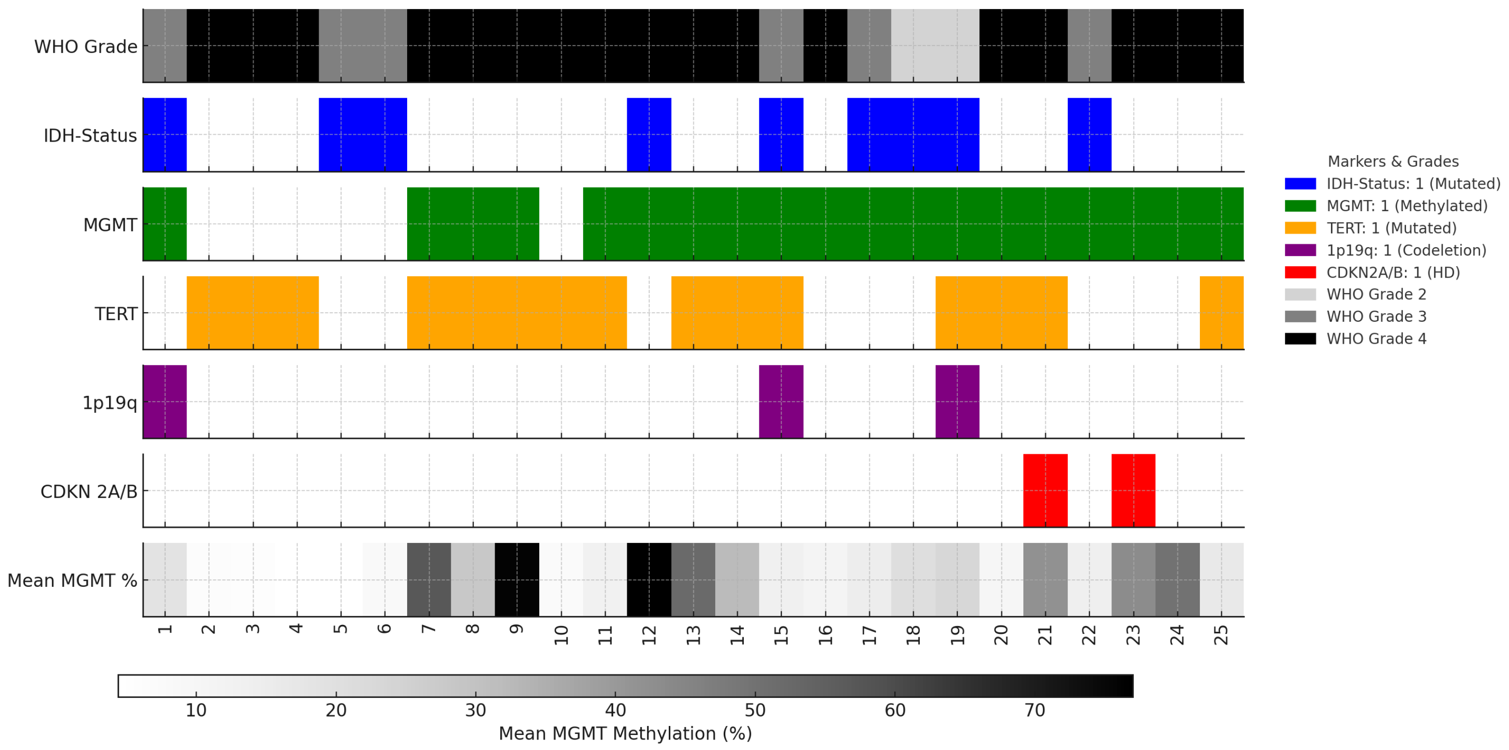

Supplement: Supplementary file 3 — Supplementary Material 3 [file 11060_2025_5340_MOESM3_ESM.pdf]
