# Supplementary material for: GLIMMER: an interim subgroup analysis from an ongoing prospective study evaluating hyperspectral imaging for MGMT promoter methylation in gliomas
Source: J Neurooncol. 2025 Nov 17;176(1):86. doi: 10.1007/s11060-025-05340-2 (PMC12628469; doi:10.1007/s11060-025-05340-2)

Supplementary figure 4. Diagnostic performance of HSI parameters for predicting IDH-1 mutation.
Receiver operating characteristic (ROC) curves of hyperspectral imaging (HSI) parameters—near-infrared (NIR), optical homogeneity index (OHI), tissue oxygenation (StO_2), and tissue water index (TWI)—for discrimination of IDH-1 mutant versus wild-type gliomas. Corresponding cut-off values, AUCs with 95% confidence intervals, sensitivities, and specificities are indicated.

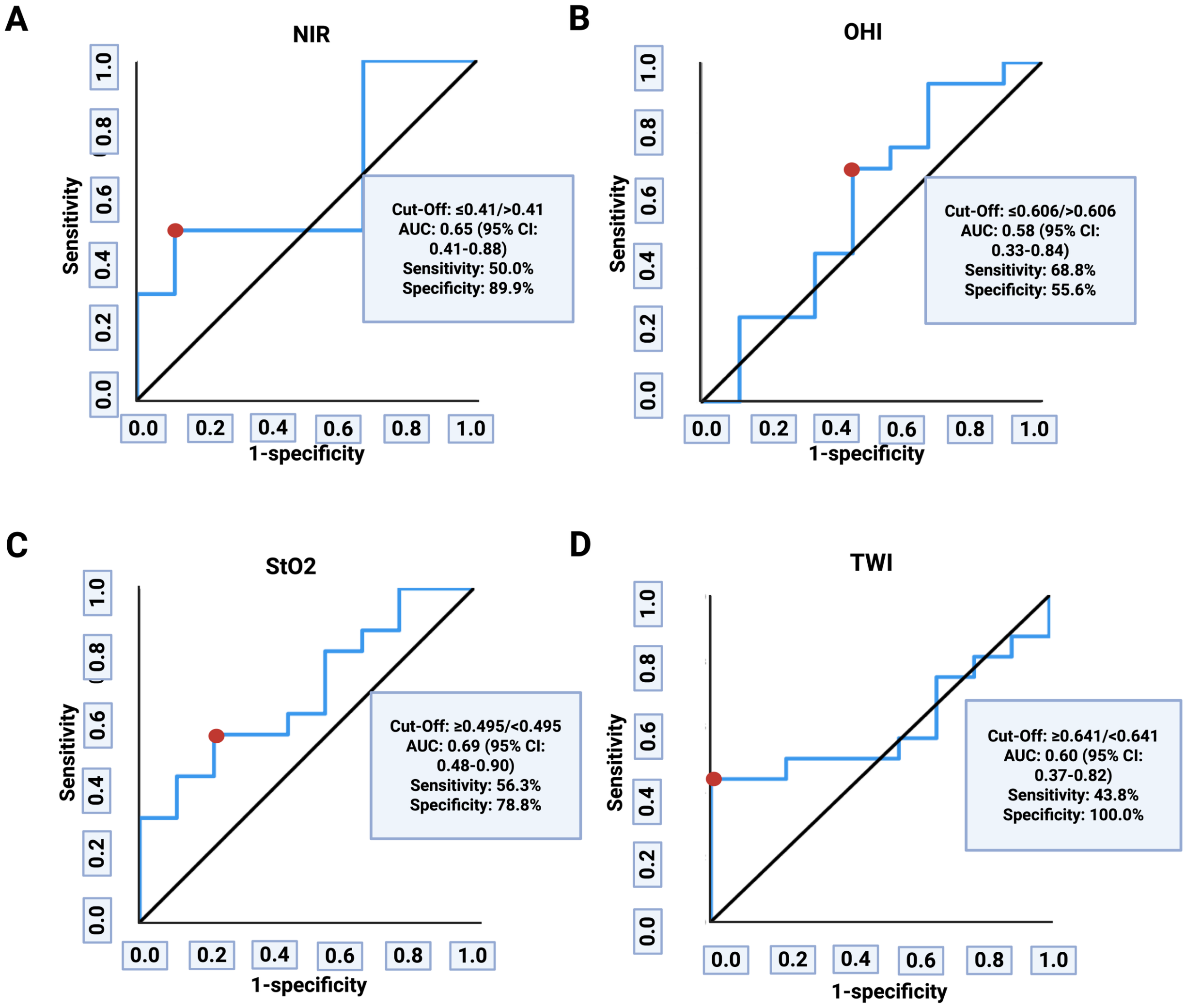

Supplement: Supplementary file 4 — Supplementary Material 4 [file 11060_2025_5340_MOESM4_ESM.pdf]
