# Supplementary material for: GLIMMER: an interim subgroup analysis from an ongoing prospective study evaluating hyperspectral imaging for MGMT promoter methylation in gliomas
Source: J Neurooncol. 2025 Nov 17;176(1):86. doi: 10.1007/s11060-025-05340-2 (PMC12628469; doi:10.1007/s11060-025-05340-2)

Supplementary Figure 5. Diagnostic performance of HSI parameters for predicting TERT promoter mutation.
ROC curves illustrating the discriminative ability of individual HSI parameters (NIR, OHI, StO_2, and TWI) for TERT promoter mutation status. Optimal cut-offs and diagnostic metrics (AUC, 95% CI, sensitivity, and specificity) are displayed within each panel.

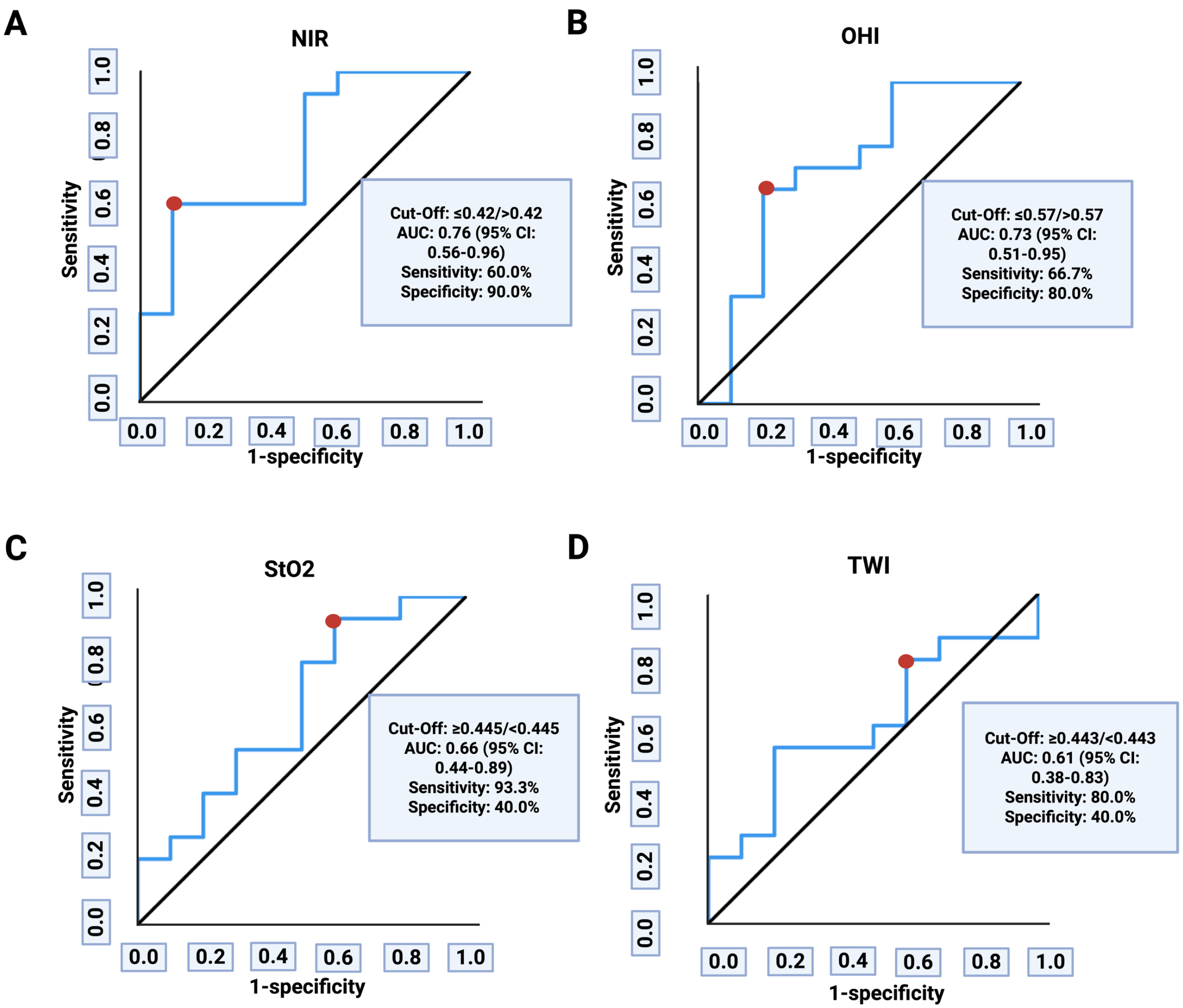

Supplement: Supplementary file 5 — Supplementary Material 5 [file 11060_2025_5340_MOESM5_ESM.pdf]
