# Supplementary material for: GLIMMER: an interim subgroup analysis from an ongoing prospective study evaluating hyperspectral imaging for MGMT promoter methylation in gliomas
Source: J Neurooncol. 2025 Nov 17;176(1):86. doi: 10.1007/s11060-025-05340-2 (PMC12628469; doi:10.1007/s11060-025-05340-2)

Supplementary Figure 6. Diagnostic performance of continuous OHI + TWI model for predicting MGMT promoter methylation.
ROC curve of the logistic regression model using continuous OHI and TWI values as predictors of MGMT methylation. The model achieved an AUC = 0.86 (95% CI 0.69–1.00), sensitivity = 84.2%, and specificity = 83.3%. The shaded area represents the 95% confidence interval.

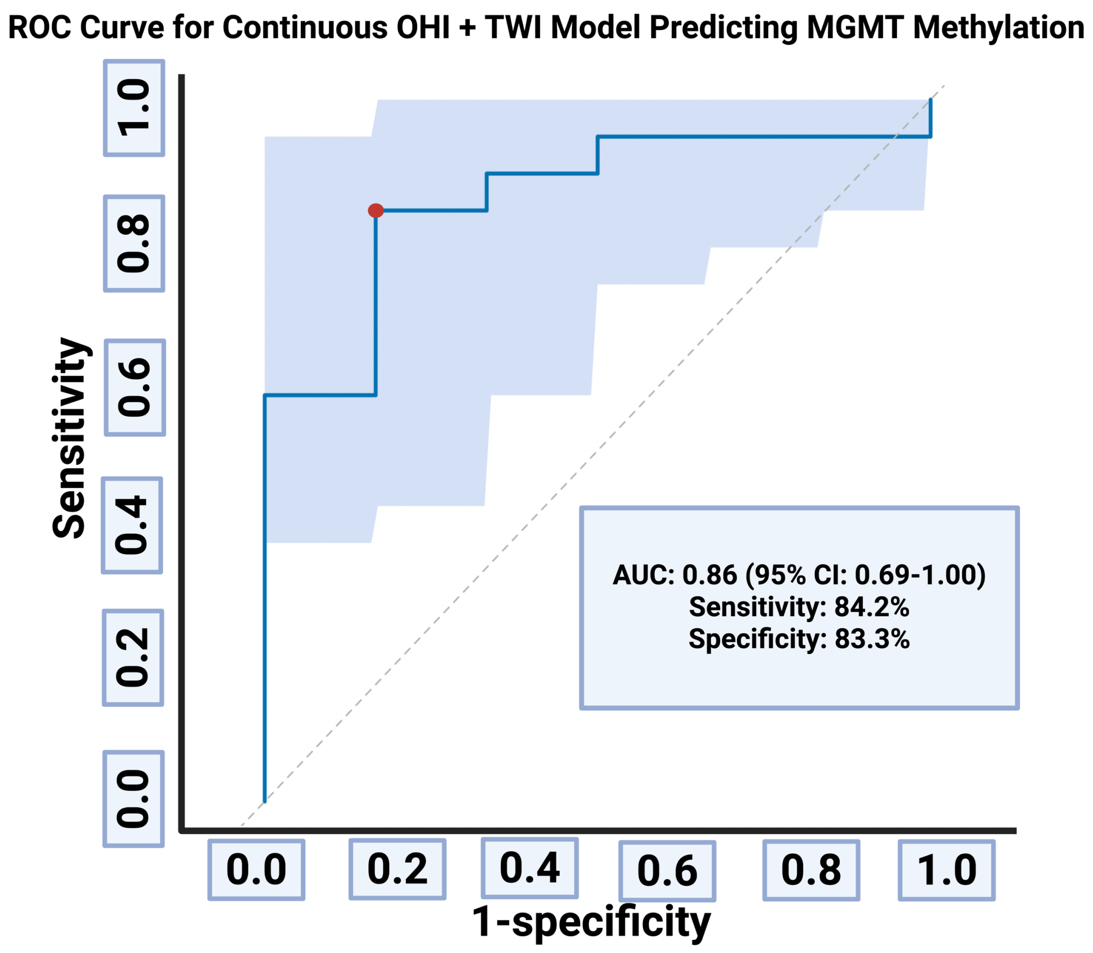

Supplement: Supplementary file 6 — Supplementary Material 6 [file 11060_2025_5340_MOESM6_ESM.pdf]
