# Supplementary material for: GLIMMER: an interim subgroup analysis from an ongoing prospective study evaluating hyperspectral imaging for MGMT promoter methylation in gliomas
Source: J Neurooncol. 2025 Nov 17;176(1):86. doi: 10.1007/s11060-025-05340-2 (PMC12628469; doi:10.1007/s11060-025-05340-2)
Supplement: Supplementary file 7 — Supplementary Material 7 [file 11060_2025_5340_MOESM7_ESM.pdf]

Clinic and Polyclinic for Neurosurgery
University Hospital Leipzig
Head: Prof. Dr. med. Erdem Güresir
Phone +49 341 97 17500
Fax +49 341 97 17509

Innovation Center Computer Assisted Surgery (ICCAS)
University of Leipzig, Faculty of Medicine
Director: Prof. Dr. med. Andreas Melzer
Phone +49 341 97 12000
Fax +49 341 97 12009

05.02.2024, V1

Application for submission to the Ethics Committee of the Medical Faculty of the University of Leipzig

"Development of a spectral tissue library and perfusion imaging using hyperspectral imaging (HSI) in intracranial vascular and neoplastic pathologies"

(short: HSI tissue library and perfusion imaging in cranial neurosurgery)

Attachments to the application form for prospective study projects of an interventional or purely observational nature

Applicant

Dr. med. Johannes Wach, MBA
Clinic and Polyclinic for Neurosurgery
University Hospital Leipzig, AöR
Liebigstraße 20
04103 Leipzig

Phone +49 341 97 17500
Fax +49 341 97 17509
Email johannes.wach@medizin.uni-leipzig.de

1 Synopsis of the project

1.1 Background

Perfusion imaging is a relevant part of perioperative and intraoperative imaging for neuro-oncological diseases (1). In the intraoperative setting, fluorescence-based procedures are mainly used to optimize the extent of resection (2). However, the high-quality evidence confirmed by a phase III study only applies to the use of 5-aminolevulinic acid in primary malignant gliomas (3). For other tumor pathologies of the central nervous system (CNS), such as recurrent gliomas, brain metastases and meningiomas, there is currently no intraoperative imaging method established with high-quality evidence (levels 1 and 2). Hyperspectral imaging (HSI) is an emerging optical imaging technique with the potential to improve intraoperative tissue detection and diagnostics. In contrast to other intraoperative imaging procedures, no ionizing radiation or medication or fluorescent dyes are applied, so that one can speak of a non-invasive procedure. HSI has already been studied in a wide range of other surgical specialties (4). HSI could be a future imaging method in the intraoperative differentiation of pathological and healthy tissue. Applications have already been made in the context of case series in the field of skull base tumor surgery and in neurovascular operations such as bypass surgery for the measurement of cerebral cortical perfusion (5,6). However, there is currently no established data with histopathological correlation.

1.2 Objectives

The following study is therefore intended to be a prospective observational study that investigates the feasibility of using HSI to visualize perfusion and tissue classification in gliomas, brain metastases, meningiomas and vascular pathologies (cavernoma, arteriovenous malformation, aneurysm).

1.3 Methodology

For ethical and functional reasons, the collection of control samples from putative healthy brain tissue is not possible and is not planned, so that patients in this study will not receive a non-standard therapy. Intraoperatively, the tumor tissue is imaged using a CE-approved HSI camera. The tumor pathologies and parts from various areas (tumor center, necrosis, cyst, solid part), which have already been removed by microsurgery and navigation, are displayed again by the HSI camera after the resection, which has already been carried out with standard methods (microsurgery & neuronavigation), before being sent to the neuropathology department. The HSI datasets before and after resection will be examined with histopathological properties such as tumor cell proliferation [Ki-67 or Molecular Immunology Borstel-1 (MIB-1) index], molecular properties in gliomas [methyl-guanine DNA methyltransferase (MGMT) & isocitrate dehydrogenase (IDH)] and other immunohistochemical markers prescribed by the WHO for the diagnosis of CNS tumors (7). Clinical data such as demographic parameters, medication, type of tumor or previous treatment (e.g. radiation) are to be recorded pseudonymously. For this purpose, clinical data is collected from the SAP medical record system in the SPSS version 29 software for Windows. The data is stored pseudonymously on a password-protected server of the Clinic for Neurosurgery. For further spectral analysis, the pseudonymized HSI and clinical data are transmitted to the Innovation Center Computer Assisted Surgery. Data will not be passed on to the camera manufacturer (Diaspective Vision GmbH, Am Salzhaff-Pepelow) under any circumstances.

1.4 Cohort

For the study project, all patients who have to undergo microsurgical surgery for a glioma, brain metastases or meningioma are to be included.

1.5 Duration

The study is expected to recruit for a total of 24 months. The enrollment of 100-120 patients is expected during this period. It is an observational feasibility study and therefore there is an exploratory approach. The results will be presented by means of univariate analyses (Fisher's exact test (two-sided) for categorical variables, Pearson's correlation) and frequency tables.

2 Protocol

2.1 General study information

Title	Establishment of a spectral tissue library and perfusion imaging using hyperspectral imaging (HSI) in intracranial vascular and neoplastic pathologies
Lemma	HSI Tissue Library and Perfusion Imaging in Cranial Neurosurgery
Registration	German Clinical Trials Register
Study Investigators (Name, Title, Responsibilities)	Dr. med. Johannes Wach, MBA (Director of Studies, Data Collection and Analysis)<br>Prof. Dr. med. Erdem Güresir (Deputy Director of Studies)
Place of study (name, address, phone)	Clinic and Polyclinic for Neurosurgery<br>University Hospital Leipzig, AöR<br>Liebigstraße 20, 04103 Leipzig<br>Phone +49 341 97 17500
Participating laboratories, institutes, departments (names, addresses)	Innovation Center Computer Assisted Surgery (ICCAS), Research Group Intraoperative Multimodal Imaging<br>Faculty of Medicine, University of Leipzig<br>Prof. Dr. med. Andreas Melzer<br>• Prof. Dr. med. Andreas Melzer (Head of Institute)<br>• M. Sc. Annekatrin Pfahl (Spectral Data Analysis)<br>• M. Sc. Hannes Köhler (Spectral Data Analysis)<br>Semmelweisstraße 14, 04103 Leipzig
Doctoral thesis (name, desired title)	In planning (Dr. med.)
Financing your studies	Budget (Cost Center: 72211000)
Legal basis	§15 BO SLÄK, GDPR, Declaration of Helsinki

2.2 State of research incl. derivation of scientific questions

2.2.1 Hyperspectral Imaging (HSI)

Hyperspectral imaging (HSI) is based on the analysis of the light reflected by the tissue. For this purpose, the area to be investigated is illuminated with harmless light, usually in the visible and near-infrared range. The incident light undergoes a variety of scattering and absorption processes, the latter through the body's own chromophores such as melanin, hemoglobin or water depending on the wavelength of the incident light. Depending on the composition of the skin area, i.e. the distribution of chromophores within the skin area, the proportion of light per wavelength that is absorbed or scattered back to the skin surface varies. By analyzing these light components per wavelength, e.g. with a spectrometer or optical filters in combination with high-resolution camera systems, it is then possible to infer the presence of certain chromophores and thus the (anomalous) tissue composition. (8) This modality does not require the administration of a contrast agent and is completely contact-free.

2.2.2 HSI in Neurosurgery

In neurosurgery, HSI has been used in recent years mainly to differentiate tumorous and healthy tissue. The Institute of Applied Microelectronics of the University of Las Palmas on Gran Canaria demonstrated in various works in the EU project HELICoiD[1] the feasibility of a specially designed HSI system covering the spectral range of 400-1700 nm. They justify the intraoperative application of HSI in the advantages over alternatives such as neuro-navigation, intraoperative magnetic resonance imaging (iMRI) or 5-ALA fluorescence imaging. These include faster data collection and higher spatial resolution compared to iMRI and imaging without the administration of contrast agents (5-ALA) and associated side effects, as well as the possible differentiation of multiple tissue types. (9) The combined application of supervised and unsupervised machine learning techniques allowed the delineation of glioblastoma, blood vessels, healthy tissue and background in five patients, with the spectral similarity of blood vessels or blood to the tumor tissue leading to some false-positive results (10). Later, Fabelo et al. compared the method with classification results of neural networks in six patients with glioblastoma. Despite significantly increased sensitivity and accuracy when using neural networks in the binary classification case (tumor vs. healthy tissue), an error rate of 58% remained in the multiclass case. This means that 58% of the tumor pixels were classified as "non-tumor". (11) In individual cases, an improvement could be achieved by reducing the analyzed spectral ranges (12).

Urbanos et al. also pursued the approach of a significantly reduced number of spectral information and machine learning methods. The data collection was carried out here with a XIMEA snapshot camera[2], which provides 24 spectral information in the range of 665-960 nm. The image data of twelve patients included grade III-IV gliomas, healthy tissue, arterial and venous blood, and dura mater. The accuracy across all classes was 60%. (5)

Hao et al. built on the results and datasets of the HELICoiD project and combined various deep learning methods to further improve glioblastoma identification. Across all classes, the accuracy of 80% (11) to 97%. (13) Nevertheless, the small number of patients remains a limitation of this study.

---

[1] HypErspectraL Imaging Cancer Detection | HELiCoiD | Project | Fact sheet | FP7 | CORDIS | European Commission (europa.eu)
[2] XIMEA - Hyperspectral Snapshot USB3 camera 24 bands 665-960nm

With the aim of simplifying and accelerating intraoperative data collection and identification of gliomas, Puustinen et al. presented a new microneurosurgical HSI system consisting of a Senop snapshot camera[3], a tunable light source[4] and a Zeiss surgical microscope[5], incl. clinical workflow in a case study (third-degree glioma) (14). With the help of this integrated system, the surgical process for intraoperative imaging does not have to be interrupted. The lighting can be adapted to the situation at hand (white light, fluorescence imaging or HSI). However, the selected spectral range and the classification algorithms need to be evaluated in extensive clinical trials.

All these studies share the small number of patients included, relatively high misclassifications and a low consensus on the spectral ranges to be used for a fast and correct tissue classification in the future. The data collection at our clinic is therefore intended to verify the results of previous research and to build on them in order to improve tissue differentiation using HSI.

2.3 Literature

1. Verburg N, de Witt Hamer PC. State-of-the-art imaging for glioma surgery. Neurosurg Rev. 1 June 2021; 44(3):1331–43.
2. Naik A, Smith EJ, Barreau A, Nyaeme M, Cramer SW, Najafali D, et al. Comparison of fluorescein sodium, 5-ALA, and intraoperative MRI for resection of high-grade gliomas: A systematic review and network meta-analysis. Journal of Clinical Neuroscience. 1 April 2022; 98:240–7.
3. Stummer W, Pichlmeier U, Meinel T, Wiestler OD, Zanella F, Reulen HJ. Fluorescence-guided surgery with 5-aminolevulinic acid for resection of malignant glioma: a randomised controlled multicentre phase III trial. The Lancet Oncology. 1 May 2006; 7(5):392–401.
4. Barberio M, Benedicenti S, Pizzicannella M, Felli E, Collins T, Jansen-Winkeln B, et al. Intraoperative Guidance Using Hyperspectral Imaging: A Review for Surgeons. Diagnostics. November 2021; 11(11):2066.
5. Urbanos G, Martin A, Vazquez G, Villanueva M, Villa M, Jimenez-Roldan L, et al. Supervised Machine Learning Methods and Hyperspectral Imaging Techniques Jointly Applied for Brain Cancer Classification. Sensor. June 2021; 21(11):3827.
6. Mori M, Chiba T, Nakamizo A, Kumashiro R, Murata M, Akahoshi T, et al. Intraoperative visualization of cerebral oxygenation using hyperspectral image data: a two-dimensional mapping method. Int J CARS. 1 November 2014; 9(6):1059–72.
7. Louis DN, Perry A, Wesseling P, Brat DJ, Cree IA, Figarella-Branger D, et al. The 2021 WHO Classification of Tumors of the Central Nervous System: a summary. Neuro-Oncology. 1 August 2021; 23(8):1231–51.
8. Lu G, Fei B. Medical hyperspectral imaging: a review. JBO. January 2014; 19(1):010901.
9. Fabelo H, Ortega S, Lazcano R, Madroñal D, M. Callicó G, Juárez E, and Others An Intraoperative Visualization System Using Hyperspectral Imaging to Aid in Brain Tumor Delineation. Sensor. February 2018; 18(2):430.
10. Fabelo H, Ortega S, Ravi D, Kiran BR, Sosa C, Bülters D, and others Spatio-spectral classification of hyperspectral images for brain cancer detection during surgical operations. PLOS ONE. 19 March 2018; 13(3):E0193721.
11. Fabelo H, Halicek M, Ortega S, Shahedi M, Szolna A, Piñeiro JF, et al. Deep Learning-Based Framework for In Vivo Identification of Glioblastoma Tumor using Hyperspectral Images of Human Brain. Sensor. January 2019; 19(4):920.
12. Martinez B, Leon R, Fabelo H, Ortega S, Piñeiro JF, Szolna A, and others Most Relevant Spectral Bands Identification for Brain Cancer Detection Using Hyperspectral Imaging. Sensor. January 2019; 19(24):5481.
13. Hao Q, Pei Y, Zhou R, Sun B, Sun J, Li S, and others Fusing Multiple Deep Models for In Vivo Human Brain Hyperspectral Image Classification to Identify Glioblastoma Tumor. IEEE Transactions on Instrumentation and Measurement. 2021; 70:1–14.
14. Puustinen S, Vrzáková H, Hyttinen J, Rauramaa T, Fält P, Hauta-Kasari M, et al. Hyperspectral Imaging in Brain Tumor Surgery—Evidence of Machine Learning-Based Performance. World Neurosurgery. 1 July 2023; 175:E614–35.

[3] HSC-2 Hyperspectral Camera 500-900nm - Senop
[4] Spectra Tune Lab - Ledmotive
[5] ZEISS OPMI PENTERO 800 – The multidisciplinary surgical microscope

15. Strand PS, Berntsen EM, Fyllingen EH, Sagberg LM, Reinertsen I, Gulati S, et al. Brain infarctions after glioma surgery: prevalence, radiological characteristics and risk factors. Acta Neurochir. 1 November 2021; 163(11):3097–108.
16. White ML, Zhang Y, Yu F, Shonka N, Aizenberg MR, Adapa P, et al. Post-operative perfusion and diffusion MR imaging and tumor progression in high-grade gliomas. PLOS ONE. 18 March 2019; 14(3):E0213905.
17. Alexander MD, Connolly ES, Meyers PM. Revisiting normal perfusion pressure breakthrough in light of hemorrhage-induced vasospasm. World Journal of Radiology. 28 June 2010; 2(6):230–2.
18. De la Garza-Ramos R, Kerezoudis P, Tamargo RJ, Brem H, Huang J, Bydon M. Surgical complications following malignant brain tumor surgery: An analysis of 2002–2011 data. Clinical Neurology and Neurosurgery. 1 January 2016; 140:6–10.

2.4 Study Objectives

2.4.1 Primary study objectives

In this project, the possibilities and potential of using non-invasive imaging optical-spectrometric measurement technology for the differentiation of tissue types (meningioma, glioma, brain metastasis versus healthy tissue) and physiological tissue parameters (perfusion, blood-brain barrier disorder) will be investigated in order to be able to perform a non-invasive intraoperative tissue analysis in the future.

For this purpose, measurement data will be collected from suitable intraoperative images by means of newly developed spectral optical imaging measurement methods (so-called hyperspectral cameras). These are used to evaluate the possibility of determining more objective and meaningful parameters in comparison with the tissue that has already been resected and the usual histological results of the specimens taken (only if there is an existing medical indication), clinical evaluations and/or established reference methods. The localization of the developed perfusion and tissue maps can be traced back for correlation analysis with the removed tissue (only with an existing medical indication) by means of screenshots by neuronavigation (standard tool for resection of brain tumors).

In particular, it will be investigated whether objective parameters can be determined for a quantifiable description of the various tissues (gliomas, metastases, meningiomas) that are comparable in their significance to a histological analysis. The imaging measurement data developed by HSI can be correlated with the entity-specific immunohistochemical and molecular markers (collected as standard for diagnosis). In gliomas, these include the following parameters: Ki-67 /Molecular Immunology Borstel-1 (MIB-1) index, molecular properties in gliomas [methyl guanine DNA methyltransferase (MGMT), telomerase reverse transcriptase (TERT) promoter status, isocitrate dehydrogenase (IDH)], ATRX expression, 1p19q codeletion. All other histopathological and molecular parameters for gliomas and meningiomas correspond to the standardized diagnostics required by the WHO for the classification of brain tumors (7). To this end, the chemical composition of the analyzed tumor tissue samples will be determined primarily from the measurement data.

In this project, a novel measurement method for clinical use is to be investigated, the available measurement systems are used exclusively for data acquisition within the framework of the project. This is not a clinical trial of the devices used.

2.4.2 Secondary study objectives

After neurosurgical tumor interventions, cerebral infarctions can occur, which may be relevant for the prognosis. In a prospective study, infarctions could be identified after 44% of the procedures (15). In addition to the focal neurological deficits that arise in the short term, especially from this, the infarctioned and thus both perfusion and diffusion disorders also seem

to be related to tumor progression (16). Especially after the resection of vascular pathologies (cavernomas, arteriovenous malformation), the perfusion of healthy tissue can change significantly after removal of pathological vascular malformations (17).

2.4.3 Primary targets

**Clinical data**	**Description**
Correlation with new postoperative deficits (motor, sensitive, language, cranial nerve deficits, epilepsy)	The measurement data from perfusion imaging will be correlated with newly developed postoperative deficits (paresis, hypoesthesia, cranial nerve palsy, frequency and type of epileptic seizures)
Correlation with demographic parameters	The measurement data from perfusion imaging will be correlated with new demographic parameters (age, gender)
Correlation with diagnostic histopathological markers	The measurement data from perfusion imaging will be correlated with the histopathological tumor characteristics collected as standard for diagnosis (e.g., proliferation rate, density of inflammatory cell infiltrates (macrophages)
Correlation with diagnostic molecular markers	The measurement data from perfusion imaging are to be correlated with the molecular tumor properties collected as standard for diagnosis (e.g. in gliomas: IDH-1 status, MGMT promoter methylation)

**HSI Data**	**Description**
Tissue oxygenation (StO2)<br>see Fig. 1a	Relative O2 saturation of the tissue in the microcircular system in superficial tissue layers (penetration depth: approx. 1 mm)
Tissue Hemoglobin Index<br>*(Tissue haemoglobin index = THI)*<br>see Fig. 1b	Existing hemoglobin distribution in the microcircular system of the tissue area under consideration (index value)
Near-Infrared Perfusion Index<br>*(Near-InfraRed = NIR Perfusion Index)*<br>see Fig. 1c	Relative O2 saturation of the tissue in the microcircular system in deeper tissue layers (penetration depth: 4-6 mm) (index value)
Fabric Water Index<br>*(Tissue Water Index = TWI)*<br>see Fig. 1d	Existing water distribution in the tissue area under consideration (index value)
HSI Raw Data	Reflectance values of the tissue area under consideration

**Reference**	**Description**
Histopathological findings	Proliferation: Ki-67/MIB-1 Index<br>Molecular genetics: IDH-1, MGMT, CDKN2A/B, TERT promoter mutation

Neuronavigation	Comparison of the anatomical localization in the MRI with the localization of the perfusion measurements using screenshots from neuronavigation
5-ALA Imaging	Correlation of perfusion measurements with the semi-quantitative (no signal, moderate, strong) 5-ALA fluorescence signal in gliomas

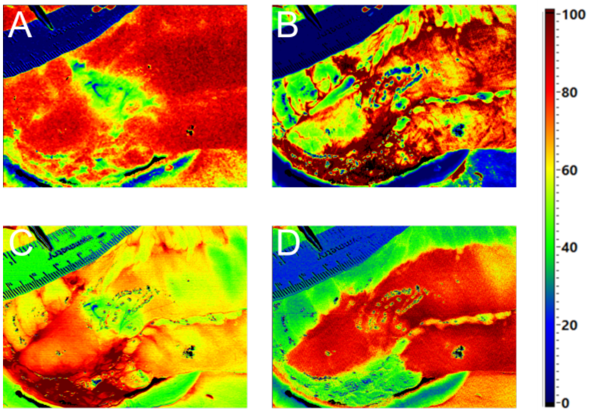

Illustration 1: HSI tissue parameters, measured at the colon.

2.4.4 Secondary Targets

Correlation with imaging postoperative infarction or bleeding and new neurological deficits within 5 days after surgery

2.4.5 Statistical objective

Exploratory

2.5 Study design

2.5.1 Character of the study

single-center, prospectively observational study

2.5.2 Expected duration of the study and scheduling of the study schedule for individuals

24 months duration of the study. The study lasts 5 days per patient (median inpatient length of stay).

2.5.3 Control groups

None

2.5.4 Randomization

None

2.5.5 Blinding

None

2.5.6 Number of study participants

100-120

2.5.7 Age

Inclusion of patients >= 18 years of age

2.5.8 Inclusion Criteria

- Patients with an intracranial mass with suspected glioma, brain metastasis or meningioma
- Patients capable of giving consent

2.5.9 Exclusion criteria with justification

- Patients unable to give consent, as voluntariness cannot be confirmed
- Pregnancy, breastfeeding women and people who wish to become pregnant, as they are particularly worthy of protection
- Children and adolescents <18 years of age, as they are particularly worthy of protection

2.5.10 Recruitment measures

Recruitment is carried out via the local university outpatient clinic with tumour consultation hours and via the interdisciplinary CNS tumour board.

2.5.11 Inclusion of persons particularly worthy of protection incl. justification and precautions

Not applicable

2.5.12 Fees for study participants

None

2.6 Procedure of the study including recording of the target variables

2.6.1 Operational process

Hyperspectral imaging is integrated into the conventional clinical environment as follows. Before the procedure (preoperative phase), interesting HSI targets (tumor necrosis, solid tumor tissue, peritumoral edema), exposure times and image sequences are defined by the team using preoperative MRI imaging. All patients receive a preoperative MRI as standard for intracranial tumor surgery, as this diagnosis is necessary for neuronavigation-assisted surgery. The HSI system is assembled and calibrated. During the procedure (intraoperative phase), the hyperspectral images are acquired according to the defined goals, but can be supplemented by interesting intraoperative tumor areas (e.g. brain invasion in meningiomas). After the procedure (postoperative period), the HSI data is analyzed along with the relevant clinical metadata, including anatomical annotations and histopathological information, MRI images, and surgical videos, and stored in our HSI database.

After the preoperative preparations, the neurosurgeon performs the intended procedure. The HSI system is moved to the surgical area, and the procedure is routinely performed using the surgical microscope. Hyperspectral imaging and visualization are controlled by a computer in a surgical tower located several meters away from the sterile field. Targeted image sequences are used to acquire hyperspectral data with or without fluorescent contrast agents in still images and video rates. The surgical microscope's built-in camera captures red, green, and blue (RGB) images of the corresponding areas as anatomical references. Surgical videos are recorded and time-stamped for HSI imaging, tissue biopsies, and other relevant events. The ambient lighting remains constant during the reference shots and the entire process, with fluorescent agents only used for dimmed ceiling lights. Recording times, angles, distances and illuminations are

documented during the process. Fluorescence images as well as tissue biopsies are taken during the procedure if possible.

2.6.2 Explanation of the technology used

The TIVITA tissue camera system (Fig. 1) available at Leipzig University Hospital records the visible (VIS) and near infrared (NIR) range and the recording surface can extend over surgical areas in the neurocranium. The analysis software processes the data and generates an RGB image (red-green-blue color image, which corresponds to a normalized color image extracted from the acquired data) as well as a false-color image each for the parameters tissue oxygenation (StO2), tissue hemoglobin (THI), NIR perfusion and tissue-water index (TWI). The assessment of these parameters is carried out with the help of a camera-specific software package called TIVITA Suite. This software allows us to define defined areas on the patient's images and examine them more closely. The camera system consists of a push-broom spectrograph, which is responsible for spectral data acquisition along the x-axis, and a CMOS camera sensor, which uses a stepper motor to record the spectrograph's data in the y-direction. This creates a so-called 3D data cube. The x- and y-axes represent the spatial dimensions, with the z-axis representing the wavelengths. This results in high-resolution measurements in the spectral bandwidth between 500-1000 nm (VIS + NIR) with 100 spectral channels (distance 5nm) and a spatial resolution of 640x480 pixels. Halogen lamps or LEDs are used as lighting units. At a distance of about 50 cm from the object, the images are taken with the help of the camera. These are contact-free and possible without additional drug administrations with very fast examination times (approx. 5 s per admission), which hardly disturb or delay the surgical processes.

The declaration of conformity of the spectral cameras is attached to this letter.

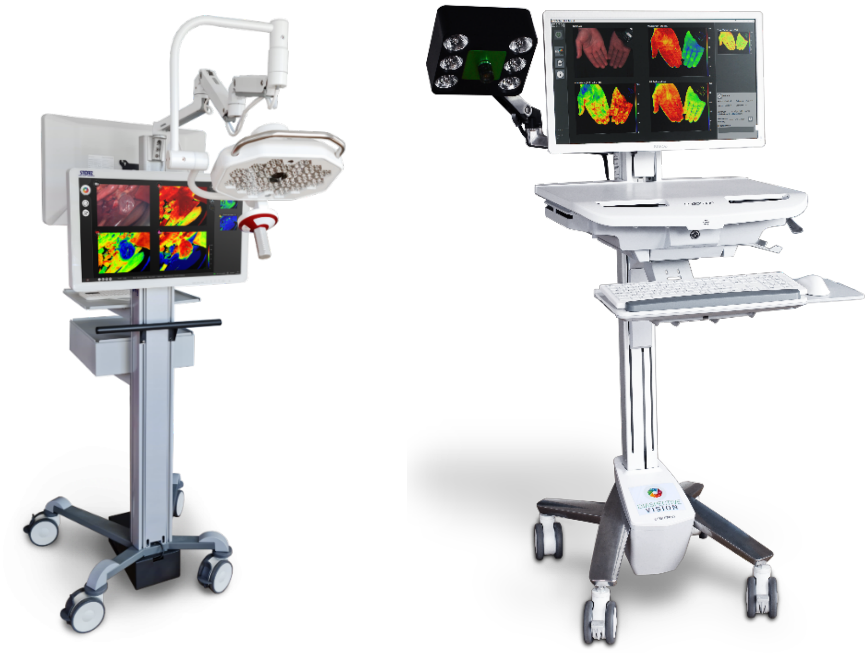

Illustration 2: The CE-approved measuring systems used in the project (TIVITA Tissue (right) and TIVITA 2.0 (left), Diaspective Vision GmbH) consisting of the hyperspectral camera with illumination unit mounted on a flexible mobile tripod, combined with a PC with special software for camera control (below the monitor).

2.7 Expected benefits for study participants

No benefit is expected for the study participants. This is an exclusively observative study.

2.8 Expected benefit for future therapies

For future patients, this study will test whether HSI is feasible in neurosurgery. Furthermore, generic correlations between HSI and tumor-specific histopathological properties will be investigated. Perfusion measurements will also be correlated with the neurological outcome and the occurrence of vascular complications (infarction, postoperative bleeding, vasogenic edema). In the long term, this could establish another intraoperative imaging tool that should help differentiate tumor tissue versus healthy CNS tissue.

2.9 Burdens and risks for study participants

Patients are subject to the risks of intracranial tumor surgery (18). HSI is used purely observatively, is contactless, non-invasive and contrast-free. The specific illumination during spectral data collection leads to a short-term (approx. 5s) heating of the observed tissue by less than 0.5 K.

2.10 List of deviations from standard treatments including justification

This is a purely observative study project. The treatment of individual patients is not affected by this.

2.11 Data processing, statistical analyses and handling of incidental findings

The data processing of pseudonymized spectral image data is carried out with the analysis software of Diaspective Vision GmbH, the TIVITA Suite, installed on the camera system. This is used to annotate recorded tissue types and structures. Further spectral analysis of the pseudonymized annotated data is carried out using Python or Matlab-based software tools developed at ICCAS.

Statistical analyses of the performance of the developed tissue classification methods are carried out using Microsoft Excel, SPSS and Python or Matlab-based software tools developed at ICCAS. The results will be presented by means of univariate analyses (Fisher's exact test (two-sided) for categorical variables, Pearson's correlation) and frequency tables. Further subgroup analyses will be carried out for the different tumor entities.

There are no additional histological examinations of the tissue that has been removed anyway independently of the study. If findings are nevertheless of a random nature, patients are referred to a family doctor.

2.12 Data management and data protection of personal data

Data collection and processing is carried out exclusively with the patient's voluntary consent in accordance with a defined list of processing activities, including a data protection impact assessment. The legal basis for the collection and processing is the GDPR.

Personal data is collected as part of clinical routine and remains on internal servers of Leipzig University Hospital (UKL). Personal data is data through which the patient can be identified by third parties. This includes, among other things, medical content, medications, examinations and treatment history. Any of the data is kept within the Department of Neurosurgery at Leipzig University Hospital under the name and date of birth. It will not be passed on to third parties under any circumstances. Locally Responsible for data management is Dr. med. Johannes Wach, MBA.

To link the spectral image data to patients, a pseudo-ID is stored in a database on the spectral camera systems. Access to it is password protected. The systems are not integrated into any network and remain at the UKL for the duration of the course. After the end of the study, all data is completely deleted from the camera systems. Data will not be passed on to Diaspective Vision GmbH at any time.

For scientific evaluation, collected image and health data (spectral data including annotations, clinical data and reference data) are passed on to the Innovation Center Computer Assisted Surgery (ICCAS) in exclusively pseudonymized form. The checklist is stored separately in the Department of Neurosurgery at Leipzig University Hospital , where it is subject to technical and organisational measures to ensure that the personal data cannot be assigned to patients by unauthorised persons.

For backup, the pseudonymized data transferred to ICCAS is uploaded to the access-restricted HSI server (hsi.iccas.de) of the University Computing Center (URZ).

Before the data is published or passed on to external parties, it is completely anonymized.

Interested parties in the study will be informed about data management and data protection before their voluntary participation. They have the right at any time to inspect the data stored about them, to obtain a free copy of them, to correct inaccurate data, to withdraw their consent and to request the deletion of data. This can be done orally or in writing without any disadvantage for them. The deletion applies to data stored within the clinic as well as data transferred to the ICCAS and data on the HSI server of the URZ.

The data will be retained for 10 years after the end or termination of the study. They are secured against unauthorized access. This is followed by complete anonymization.

2.13 Publication of results

The image data collected and analysed as well as the results of the data analysis are to be published in a completely anonymised form in conference papers, journal articles and follow-up proposals. It is also planned to make the fully anonymised data available in the form of a research dataset via the OpARA research data repository of Leipzig University Library.

3 Testing Laboratory Description

The Clinic and Polyclinic for Neurosurgery at Leipzig University Hospital is an established study centre. In recent years and also currently, scientific studies on the subject of neuro-oncology have been carried out continuously. Due to the excellent conditions of the UCCL and the interdisciplinary CNS tumor board, there is a high level of clinical expertise with currently about 130 interventions on gliomas, brain metastases and meningiomas annually. Modern intraoperative tools such as neuronavigation, microsurgery, intraoperative MRI and intraoperative fluorescence imaging (5-aminolevulinic acid, indocyanine green) are established.

The local clinical trial leader (Dr. med. Johannes Wach, MBA) has previous experience in conducting clinical trials. A prospective clinical trial on selective dorsal rhizotomy in children at the University Hospital Bonn has already been conducted. This study is still running bicentrically with the neurosurgery department in Leeds (UK). Furthermore, the AMG FINISHER study on the role of dexamethasone in aneurysmal subarachnoid hemorrhage is currently being conducted at Leipzig University Hospital. Dr. Wach is currently the author of

28 publications as first or last author and continues to be co-author of 23 peer-reviewed articles. The results of the planned study project will therefore be analysed and published in cooperation with the local biometrics department.

Student financing is initially provided through budget funds.

There are no conflicts of interest.

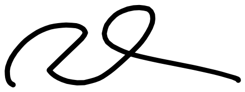

Dr. med. Johannes Wach, MBA
