# Supplementary material for: GLIMMER: an interim subgroup analysis from an ongoing prospective study evaluating hyperspectral imaging for MGMT promoter methylation in gliomas
Source: J Neurooncol. 2025 Nov 17;176(1):86. doi: 10.1007/s11060-025-05340-2 (PMC12628469; doi:10.1007/s11060-025-05340-2)
Supplement: Supplementary file 9 — Supplementary Material 9 [file 11060_2025_5340_MOESM9_ESM.docx]

| **Supplementary Table 2.** Wavelength Ranges Utilized for Hyperspectral Tissue Indices | | | |
| --- | --- | --- | --- |
| Parameter / Index | Wavelength Range 1 (nm) | Wavelength Range 2 (nm) | Spectral Region |
| Tissue Oxygenation (StO₂) | 500 – 650 | 700 – 815 | Visible + Near Infrared |
| Near-Infrared Perfusion Index (NIR-PI) | 655 – 735 | 825 – 925 | Near Infrared |
| Tissue Hemoglobin Index (THI) | 530 – 590 | 785 – 825 | Visible + Near Infrared |
| Tissue Water Index (TWI) | 880 – 900 | 955 – 980 | Near Infrared |
