# Supplementary material for: GLIMMER: an interim subgroup analysis from an ongoing prospective study evaluating hyperspectral imaging for MGMT promoter methylation in gliomas
Source: J Neurooncol. 2025 Nov 17;176(1):86. doi: 10.1007/s11060-025-05340-2 (PMC12628469; doi:10.1007/s11060-025-05340-2)
Supplement: Supplementary file 10 — Supplementary Material 10 [file 11060_2025_5340_MOESM10_ESM.docx]

| Supplementary Table 2. Patient characteristics (n = 25) | |
| --- | --- |
| Median age (IQR) (in y) | 53.0 (41.0-63.5) |
| Sex  Female  Male | 13 (52.0%)  12 (48.0%) |
| Tumor laterality  Left  Right | 6 (24.0%)  19 (76.0%) |
| Lobe  Frontal  Temporal  Parietal | 11 (44.0%)  11 (44.0%)  3 (12.0%) |
| FLAIR volume of NCE, cm^3^  median (IQR) | 80.35 (32.3-129.0) |
| T2-FLAIR intensity, median (IQR) | 1.53 (1.23-1.73) |
| ADC ratio, median (IQR) | 1.79 (1.59-2.1) |
| In-vivo HSI, median (IQR)  NIR  STO2  OHI  TWI | 0.45 (0.39-0.56)  0.51 (0.45-0.63)  0.57 (0.46-0.70)  0.56 (0.40-0.68) |
| WHO grade  2  3  4 | 2 (8.0%)  6 (24.0%)  17 (68.0%) |
| IDH  Wild-type  mutation | 16 (64.0%)  9 (36.0%) |
| MGMT promoter  Non-methylated  methylated | 6 (24.0%)  19 (76.0%) |
| TERT  Wild-type  mutation | 10 (40.0%)  15 (60.0%) |
| 1p19q codeletion  Present  Absent | 3 (12.0%)  22 (88.0%) |
| MIB-1 index, Median (IQR) | 7.0 (5.0-17.5) |
| Abbreviations: ADC, apparent diffusion coefficient; FLAIR, fluid-attenuated inversion recovery; HSI, hyperspectral imaging; IDH, isocitrate dehydrogenase; IQR, interquartile range; MGMT, O⁶-methylguanine-DNA methyltransferase; MIB-1, Molecular immunology borstel; NCE, non-contrast enhancing; NIR, near-infrared reflectance; OHI, organ hemoglobin index; STO₂, tissue oxygen saturation; TERT, telomerase reverse transcriptase; TWI, tissue water index; WHO, World Health Organization; y, years; 1p19q, chromosomal arms 1p and 19q. | |
