# Supplementary material for: GLIMMER: an interim subgroup analysis from an ongoing prospective study evaluating hyperspectral imaging for MGMT promoter methylation in gliomas
Source: J Neurooncol. 2025 Nov 17;176(1):86. doi: 10.1007/s11060-025-05340-2 (PMC12628469; doi:10.1007/s11060-025-05340-2)
Supplement: Supplementary file 11 — Supplementary Material 11 [file 11060_2025_5340_MOESM11_ESM.docx]

| **Supplementary Table 3. Group Statistics by MGMT Promoter Methylation Status** | | | | | |
| --- | --- | --- | --- | --- | --- |
| **Variable** | **MGMT Status** | **N** | **Mean** | **Std. Deviation** | **p-value** |
| **Age (years)** | Non-methylated | 6 | 52.83 | 22.57 | 0.73 |
|  | Methylated | 19 | 50.16 | 13.81 |  |
| **FLAIR Volume (cm³)** | Non-methylated | 6 | 55.69 | 66.27 | 0.18 |
|  | Methylated | 19 | 91.65 | 51.29 |  |
| **T2-FLAIR Intensity** | Non-methylated | 6 | 1.468 | 0.208 | 0.74 |
|  | Methylated | 19 | 1.510 | 0.283 |  |
| **ADC Ratio** | Non-methylated | 6 | 1.928 | 0.455 | 0.69 |
|  | Methylated | 19 | 1.844 | 0.438 |  |
| **Sex** | Non-methylated | 6 | Female: 83.3% Male: 16.7% | — | 0.16 |
|  | Methylated | 19 | Female: 42.1% Male: 57.9% | — |  |
